# Supplementary material for: Loss of the transcription factor RBPJ induces disease-promoting properties in brain pericytes
Source: Nat Commun. 2019 Jun 27;10:2817. doi: 10.1038/s41467-019-10643-w (PMC6597568; doi:10.1038/s41467-019-10643-w)
Supplement: Supplementary file 3 — Description of Additional Supplementary Files [file 41467_2019_10643_MOESM3_ESM.pdf]

Description of Additional Supplementary Information

Supplementary Data 1: **Differentially expressed genes in *Rbpj*-mutant mural cells at P7 with respect to controls.** The data file contains information on all statistically significant up- and down-regulated genes identified in P7 brain mural cells from control and *Rbpj*^{iPC} mice based on adjusted p-value <0.05 and log2-transformed log change >0.5.

Supplementary Data 2: **Differentially expressed genes in *Rbpj*-mutant mural cells at P10 with respect to controls.** The data file contains information on all statistically significant up- and down-regulated genes identified in P10 brain mural cells from control and *Rbpj*^{iPC} mice based on adjusted p-value <0.05 and log2-transformed log change >0.5.

Supplementary Data 3: **RBPJ peaks identified in pericytes.** The data file contains information on position of the 11094 RBPJ binding sites alongside with membership to clusters obtained by k-means clustering and distance to next annotated gene(s) in bp as defined by GREAT using the gene association rule 1 with default settings.

Supplementary Data 4: **List of *bona fide* RBPJ target genes in pericytes.** The data file contains 122 genes identified as RBPJ targets based on their association with RBPJ binding in their regulatory domain as defined by GREAT association rule 1 and being significantly up-regulated after loss of RBPJ in P7 *Rbpj*^{iPC} and P10 *Rbpj*^{iPC} (log2FC > 0.5 and corrected p-value < 0.05).
