# Supplementary material for: Loss of the transcription factor RBPJ induces disease-promoting properties in brain pericytes
Source: Nat Commun. 2019 Jun 27;10:2817. doi: 10.1038/s41467-019-10643-w (PMC6597568; doi:10.1038/s41467-019-10643-w)
Supplement: Supplementary file 1 — Supplementary Information [file 41467_2019_10643_MOESM1_ESM.pdf]

SUPPLEMENTARY INFORMATION

Loss of the transcription factor RBPJ induces disease-promoting properties in brain pericytes

Diéguez-Hurtado et al.

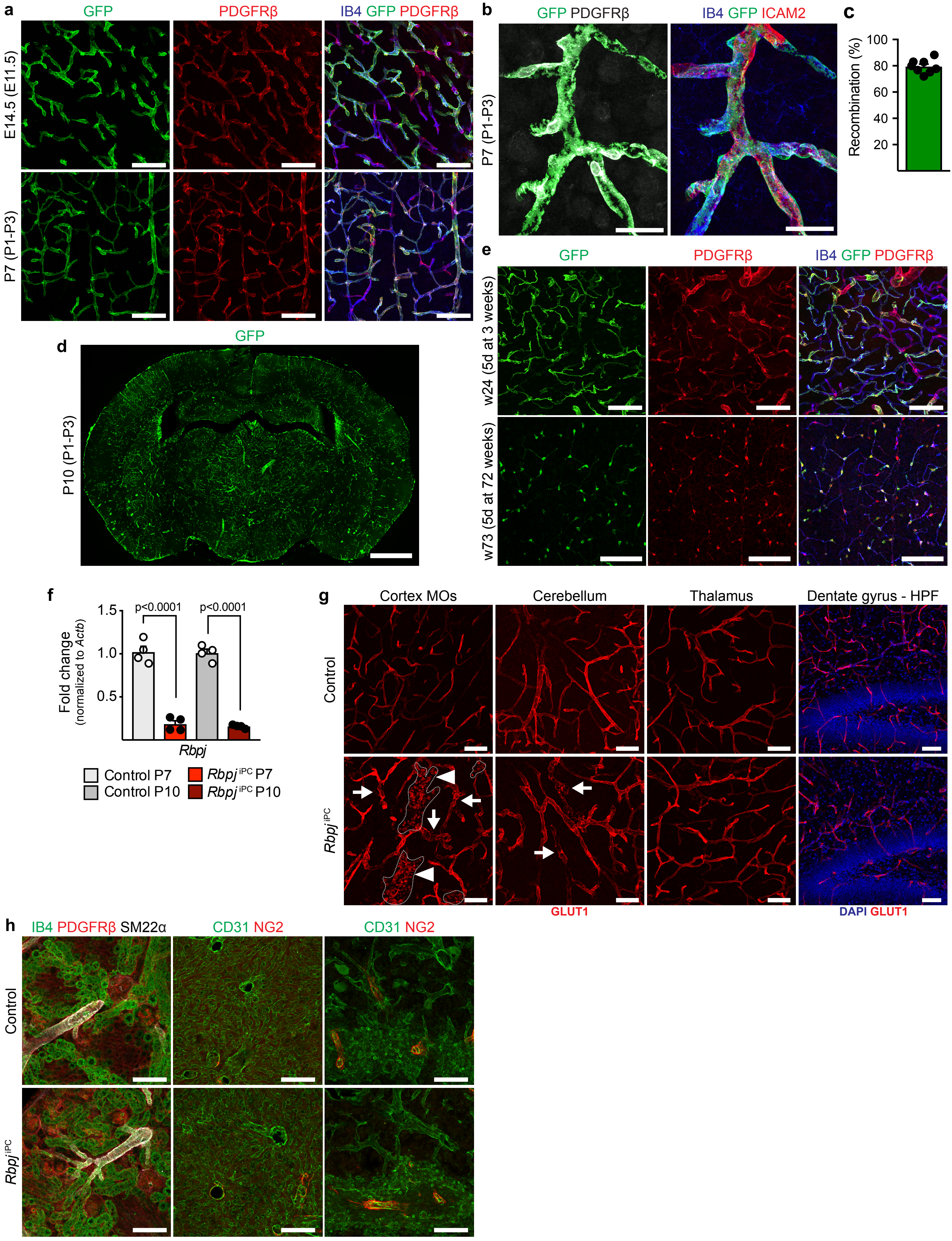
a
GFP
PDGFRβ
IB4 GFP PDGFRβ
E14.5 (E11.5)
P7 (P1-P3)
b
GFP PDGFRβ
IB4 GFP ICAM2
P7 (P1-P3)
c
Recombination (%)
100
80
60
40
20
d
GFP
P10 (P1-P3)
e
GFP
PDGFRβ
IB4 GFP PDGFRβ
w24 (5d at 3 weeks)
w73 (5d at 72 weeks)
f
Fold change
(normalized to Actb)
1.5
1.0
0.5
p<0.0001
p<0.0001
Rbpj
Control P7
Control P10
Rbpj iPC P7
Rbpj iPC P10
g
Cortex MOs
Cerebellum
Thalamus
Dentate gyrus - HPF
Control
Rbpj iPC
GLUT1
DAPI GLUT1
h
IB4 PDGFRβ SM22α
CD31 NG2
CD31 NG2
Control
Rbpj iPC

Supplementary Figure 1. Tamoxifen-inducible Cre-mediated recombination in brain mural cells and CNS-specificity of the *Rbpj*^{iPC} phenotype.

a, Confocal images of the brain cortex vasculature from E14.5 mouse embryos (top) and P7 pups (bottom) after tamoxifen induction at E11.5 or P1-P3, respectively. The *Pdgfrb-CreERT2* transgene drives efficient recombination and induces GFP (green) expression (from the *Rosa26*^{mTmG} Cre-reporter) in PDGFRβ^{+} pericytes (red) associated with IB4^{+} (blue) blood vessels. Scale bar, 100 µm.

b, High magnification images of brain cortex capillaries in *Pdgfrb-CreERT2 Rosa26*^{mTmG} P7 pups showing strict co-localization of GFP (green, as surrogate marker of recombination) and native PDGFRβ (white) expression. GFP signals are found in close vicinity of the endothelium (ICAM2, red). Scale bar, 30 µm.

c, Quantitation of recombination efficiency in P10 *Pdgfrb-CreERT2 Rosa26*^{mTmG} pups after tamoxifen induction from P1-P3.

d, Tile-scan of a coronal section from P10 *Pdgfrb-CreERT2 Rosa26*^{mTmG} brain showing homogeneous GFP expression across different brain regions. Scale bar, 1 mm.

e, Confocal images of the brain cortex vasculature from adult mice treated with tamoxifen over 5 consecutive days at 3 weeks (top) or 72 weeks (bottom) of age. GFP (green) indicates recombined pericytes (PDGFRβ^{+}, red) in blood vessels (IB4^{+}, blue). Scale bar, 100 µm.

f, Gene expression fold change of *Rbpj* in FACS-sorted P7 and P10 brain cortex mural cells. p-values, one-way ANOVA with Sidak’s test, n=4.

g, Confocal images from coronal brain sections showing blood vessels (GLUT1, red). Note vessel tortuosity (arrows) and extravasated red blood cells (RBC, GLUT1^{+}; dashed line and arrowheads) in the secondary motor area (MOs) of the *Rbpj*^{iPC} brain cortex and cerebellum. Vessel morphology is not significantly altered in the thalamus or hippocampal formation (HPF). Nuclear staining (DAPI, blue) allows identification of the dentate gyrus. Scale bar, 100 µm.

h, Confocal images from P10 kidney (left), liver (centre) and spleen (right). Blood vessels (IB4 or CD31, green) and mural cells (PDGFRβ or NG2, red) in these organs appear normal without obvious morphological changes or haemorrhages. Scale bar, 100 µm.

Error bars represent s.e.m. Source data are provided as a Source Data file.

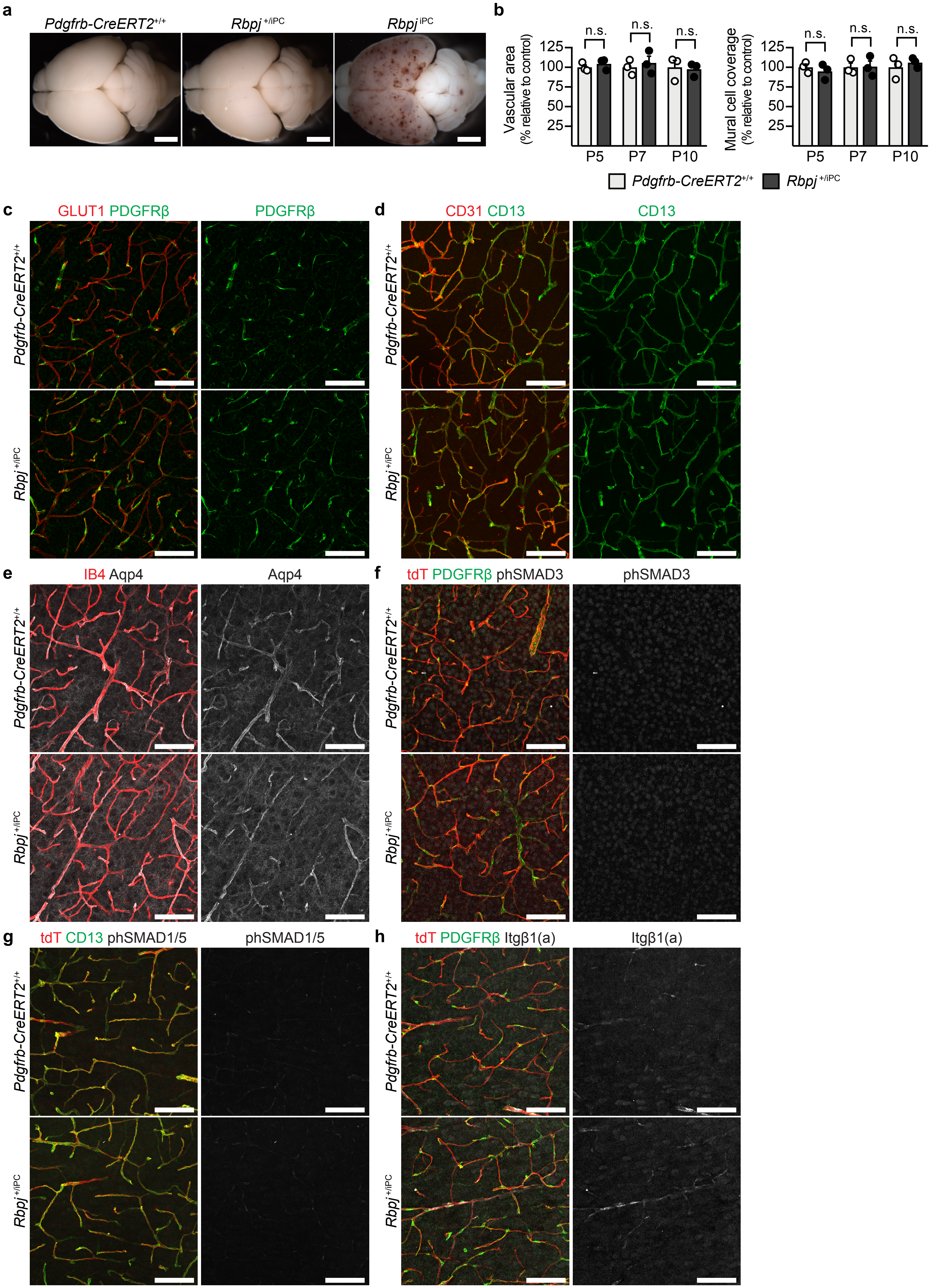

a
Pdgfrb-CreERT2+/+
Rbpj +/iPC
Rbpj iPC
b
Vascular area (% relative to control)
Mural cell coverage (% relative to control)
n.s.
125
100
75
50
25
P5
P7
P10
Pdgfrb-CreERT2+/+
Rbpj +/iPC
c
GLUT1 PDGFRβ
PDGFRβ
d
CD31 CD13
CD13
e
IB4 Aqp4
Aqp4
f
tdT PDGFRβ phSMAD3
phSMAD3
g
tdT CD13 phSMAD1/5
phSMAD1/5
h
tdT PDGFRβ Itgβ1(a)
Itgβ1(a)

Supplementary Figure 2. Hemizygous deletion of *Rbpj* in mural cells does not induce any deleterious effect in brain vascular morphogenesis.
a, Macroscopic images of whole brains from control (*PdgfrbCreERT2*$^{+/+}$) mice and littermates with hemizygous (*Rbpj*$^{+/iPC}$) or homozygous (*Rbpj*^{iP0C}) deletion of *Rbpj* at P10 following tamoxifen administration from P1-P3. Scale bar, 2 mm.
b, Quantitation of vascular area and mural cell coverage in the P5, P7 and P10 brain cortex vasculature. Note that deletion of one copy of *Rbpj* (*Rbpj*$^{+/iPC}$) does not induce any change with respect to mice with two functional copies of the gene (*PdgfrbCreERT2*$^{+/+}$). p-values, one-way ANOVA with Sidak's test, n=3.
c, **d**, Confocal images showing comparable expression of endothelial (GLUT1 in **c** and CD31 in **d**, red) and mural cell (PDGFRβ in **c**, and CD13 in **d**, green) markers in the brain vasculature of control and *Rbpj*$^{+/iPC}$ mice at P10. Scale bar, 100 μm.
e, Confocal images illustrating conserved and normal astrocyte endfeet coverage (Aqp4, white) of the brain vasculature (IB4, red) in control and *Rbpj*$^{+/iPC}$ mice at P10. Scale bar, 100 μm.
f, **g**, Confocal images showing low and comparable phosphorylation of SMAD3 (**f**, white) or SMAD1/5 (**g,** white) in the cerebrovasculature (tdT, red and PDGFRβ or CD13, green) of control and *Rbpj*$^{+/iPC}$ mice at P10. Scale bar, 100 μm.
h, Confocal images showing comparable levels of integrin β1 activation (Itgβ1(a), white) in the brain vasculature (tdT, red and PDGFRβ, green,) of control and *Rbpj*$^{+/iPC}$ mice at P10. Scale bar, 100 μm.
Error bars represent s.e.m. Source data are provided as a Source Data file.

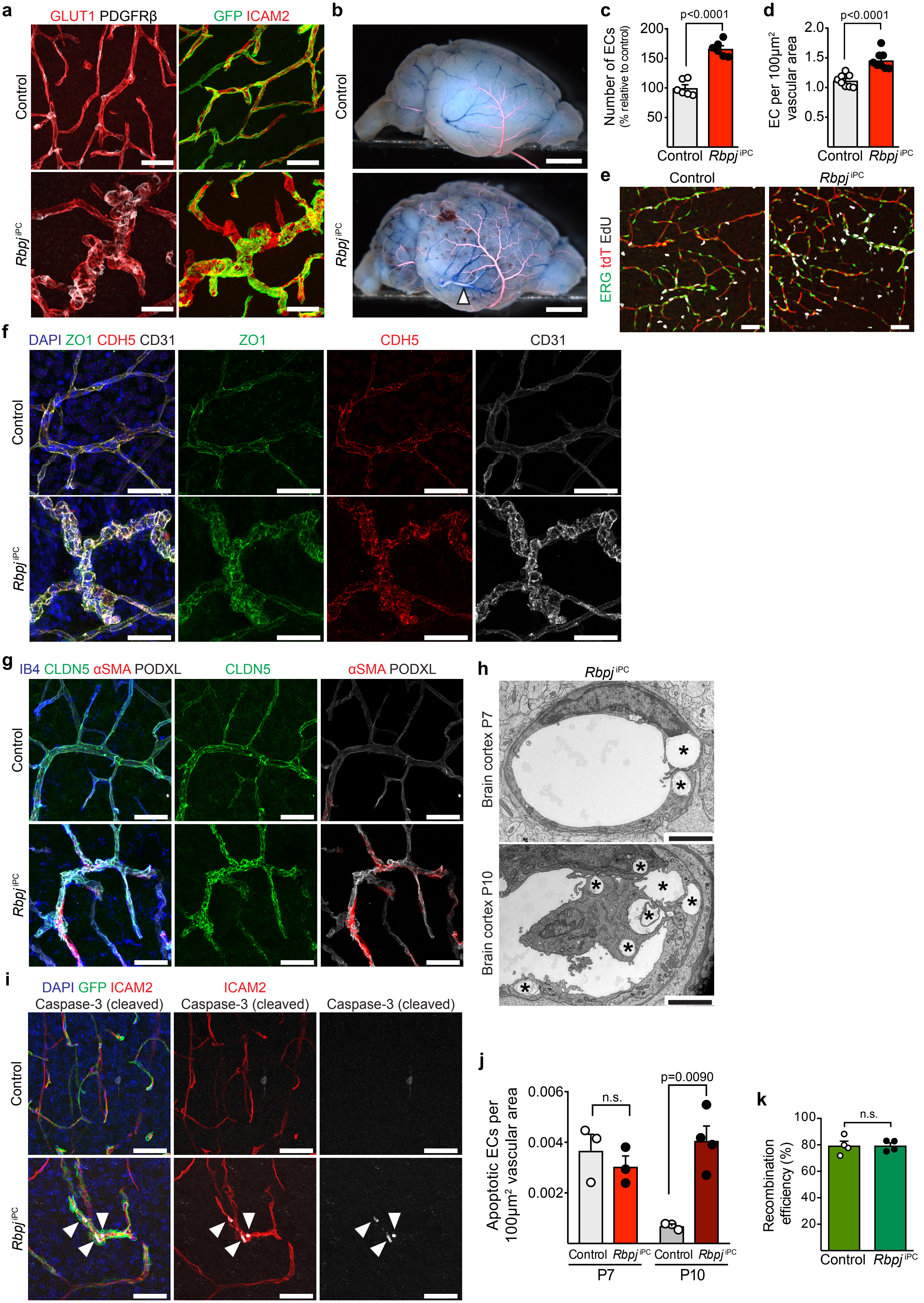
a
GLUT1 PDGFRβ
GFP ICAM2
Control
Rbpj iPC
b
Control
Rbpj iPC
c
p<0.0001
Number of ECs
(% relative to control)
200
150
100
50
Control
Rbpj iPC
d
p<0.0001
EC per 100μm²
vascular area
2.0
1.5
1.0
0.5
Control
Rbpj iPC
e
Control
Rbpj iPC
ERG tdT EdU
f
DAPI ZO1 CDH5 CD31
ZO1
CDH5
CD31
Control
Rbpj iPC
g
IB4 CLDN5 αSMA PODXL
CLDN5
αSMA PODXL
Control
Rbpj iPC
h
Rbpj iPC
Brain cortex P7
Brain cortex P10
i
DAPI GFP ICAM2
Caspase-3 (cleaved)
ICAM2
Caspase-3 (cleaved)
Caspase-3 (cleaved)
Control
Rbpj iPC
j
Apoptotic ECs per
100μm² vascular area
0.006
0.004
0.002
n.s.
p=0.0090
Control
Rbpj iPC
Control
Rbpj iPC
P7
P10
k
Recombination
efficiency (%)
100
80
60
40
20
n.s.
Control
Rbpj iPC

Supplementary Figure 3. Characterization of the *Rbpj* mutant vasculature.
a, Confocal images of brain cortex microvasculature at P10 showing severe dilation, local constrictions and ballooning. Affected blood vessels (GLUT1 or ICAM2^{+}, red) are in close association to pericytes (PDGFRβ or GFP^{+}, white and green, respectively). Scale bar, 50 µm.
b, Superficial brain vasculature from P10 mice revealed by intracardiac injection of Evans blue and pink latex. White arrowhead points to arteriovenous malformation (AVM). Detailed image of the AVM is shown in Figure **1h**. Scale bar, 2 mm.
c, **d**, Quantitation of the number of ECs per field of view at P10 (**c**) and relative number of ECs per vascular area at P7 (**d**) in control and *Rbpj*^{iPC} brain sections. p-values, Student's t-test, n=6 (c), n=8 (d).
e, Confocal images of brain cortex blood vessels labelled with tdT (red) and stained for the EC nuclear marker ERG (green) after EdU injection in P7 mice. Scale bar, 100 µm.
f, Confocal images showing comparable expression of the junction markers Zona Occludens 1 (ZO1, green) VE-Cadherin (CDH5, red) and PECAM1 (CD31, white) in P10 control and *Rbpj*^{iPC} brain vasculature. Scale bar, 50 µm.
g, Confocal images showing conserved expression of the tight junction marker Claudin 5 (CLDN5, green) in brain blood vessels (labelled with IB4, blue, and Podocalyxin, PODXL, white) of P10 mice. Note presence of αSMA^{+} pericytes (red) in *Rbpj* knockouts. Scale bar, 50 µm.
h, Electron micrographs of *Rbpj*^{iPC} brain cortex capillaries showing pinocytotic vesicles and cytoplasmic vacuolization (asterisks) in brain ECs at P7 and P10. Scale bar, 2 µm.
i, j, Confocal images (**i**) and quantitation (**j**) showing increased apoptosis (cleaved Caspase 3, white) in endothelial cells (ICAM2, red) of P10 *Rbpj*^{iPC} mice when compared to controls. Scale bar, 50 µm. p-values, Student's t-test, n=3 for analysis at P7. Welch's t-test, n=4 for analysis at P10. n.s., not statistically significant.
k, Quantitation of recombination efficiency in mural cells of control and *Rbpj*^{iPC} mice at P10. p-values, Student's t-test, n=4. n.s., not statistically significant.
Error bars represent s.e.m. Source data are provided as a Source Data file.

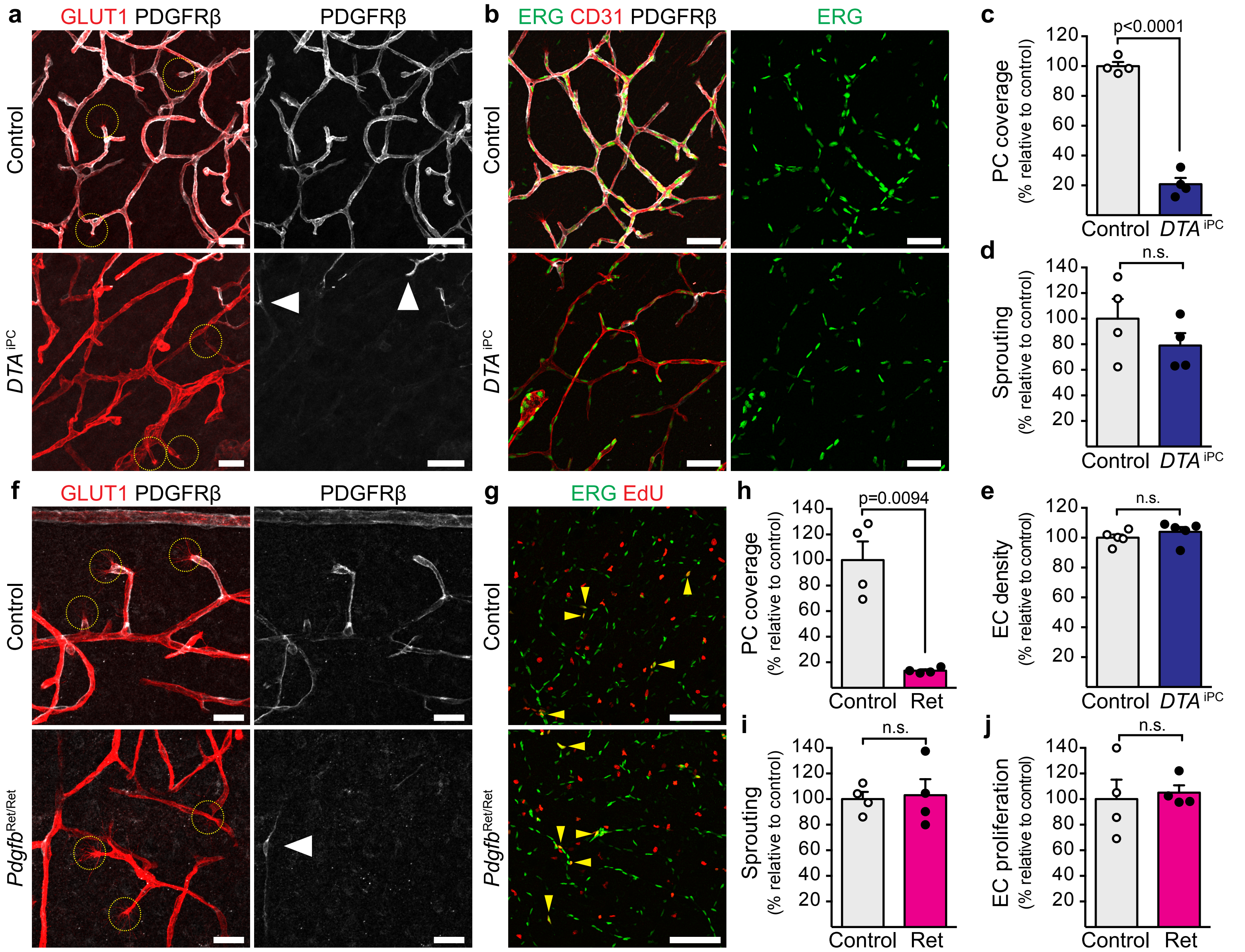

Supplementary Figure 4. Acute or chronic mural cell ablation fails to reproduce the *Rbpj*^{iPC} phenotype.

a, Confocal images of the vasculature in P6 control and *DTA*^{iPC} brain cortex (after tamoxifen injection from P1 to P3) stained for GLUT1 (red) and PDGFRβ (white). Note strong reduction in the amount of PDGFRβ$^+$ cells in *DTA*^{iPC} section (few remaining pericytes are indicated by white arrowheads) and normal sprouting (dashed circles). Scale bar, 50 µm.

b, Confocal images showing normal density of EC nuclei (ERG, green) in the vasculature (CD31, red; PDGFRβ, white) of mural cell-depleted P6 *DTA*^{iPC} animals with respect to littermate controls. Scale bar, 50 µm.

c, **d**, **e**, Quantitation of pericyte coverage (**c**), sprouting frequency (**d**), and EC density (**e**) in the P6 control and *DTA*^{iPC} brain cortex. Note that the sharp decrease in pericyte coverage has only a minor and not significant effect on the abundance of sprouts and no impact on the EC density in the *DTA*^{iPC} vasculature. p-values, Student's t-test (**c**, **d**) or Mann-Whitney U-test (**e**), n=4-5. n.s., not statistically significant.

f, Confocal images from brain cortex vasculature showing reduced pericyte (PDGFRβ, white) coverage of blood vessels (GLUT1, red) in P9 *Pdgfb*$^{\text{Ret/Ret}}$ mice relative to littermate controls. Few remaining pericytes are indicated by white arrowheads. Dashed circles indicate normal sprouting. Scale bar, 25 µm.

g, Confocal images from the P9 control and *Pdgfb*$^{\text{Ret/Ret}}$ brain cortex after EdU-labelling (red) and co-staining for EC (ERG, green). Double-positive nuclei (yellow arrowheads) are identified as proliferating ECs. Scale bar, 100 µm.

h, **i**, **j**, Quantitation of mural cell coverage (**h**), sprouting (**i**), and EC proliferation (**j**) in the P9 brain cortex of control and *Pdgfb*$^{\text{Ret/Ret}}$ animals. Despite low mural cell coverage, no significant changes are detected in sprouting angiogenesis or EC proliferation. p-values, Welch's (**h**) or Student's t-test (**i**, **j**), n=4. n.s., not statistically significant.

Error bars represent s.e.m. Source data are provided as a Source Data file.

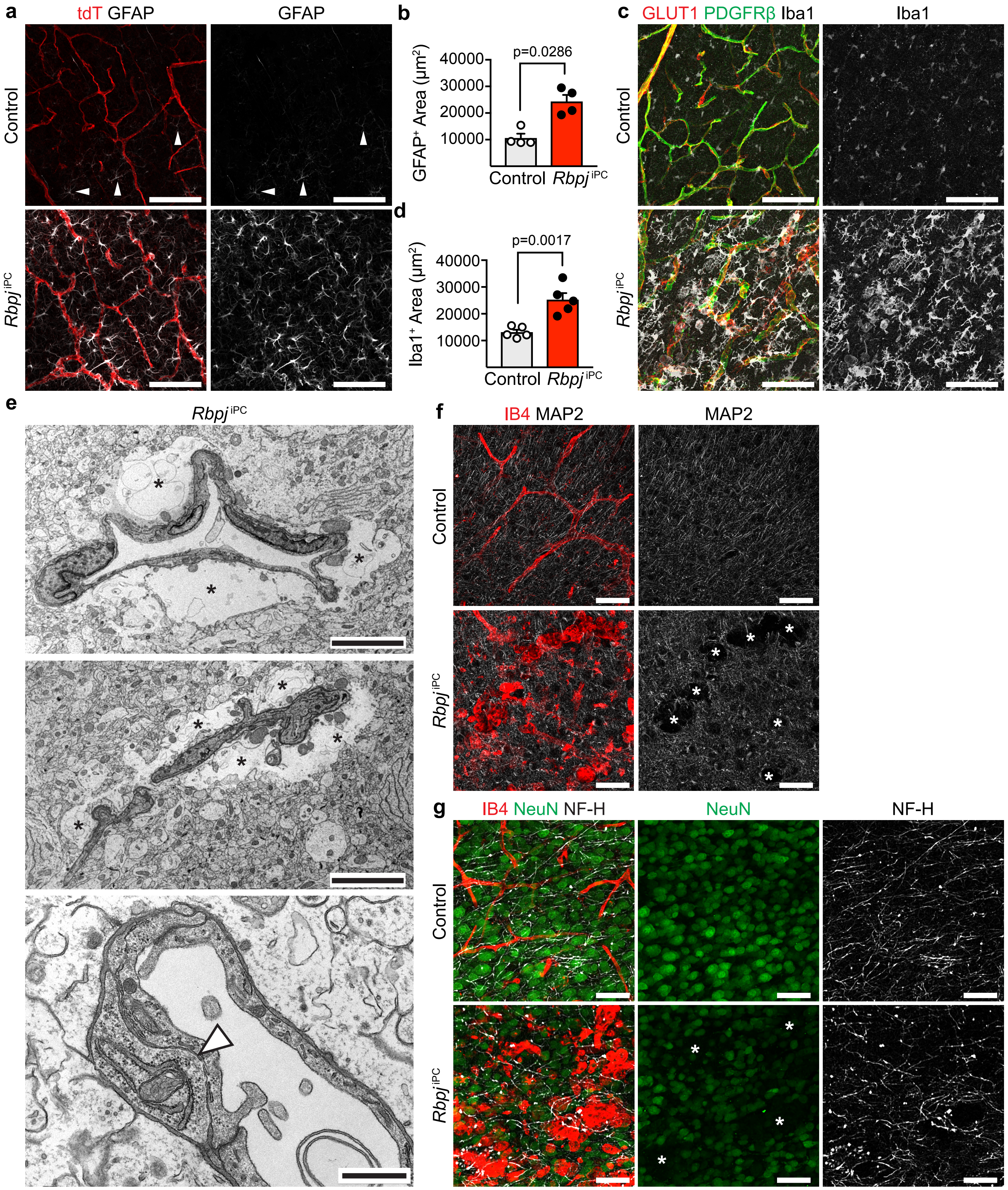

Supplementary Figure 5. *Rbpj* in pericytes is indispensable for neurovascular unit integrity.
a, Confocal images from P10 mouse brain cortices showing tdT-labelled blood vessels (red) and staining for glial fibrillary acidic protein (GFAP, white). Note strong astrogliosis in *Rbpj*^{iPC} samples, whereas GFAP is restricted to few astrocytes (white arrowheads) in control. Scale bar, 100 µm.
b, Quantitation of GFAP$^+$ area (µm^2) in the P10 control and *Rbpj*^{iPC} brain cortex. p-values, Mann-Whitney U-test, n=4.
c, Confocal images showing severe microglia (Iba1$^+$, white) infiltration and activation around the vasculature (GLUT1$^+$, red; PDGFRβ$^+$, green) in P10 *Rbpj*^{iPC} brain cortex. Scale bar, 100 µm.

d, Quantitation of Iba1$^+$ area (μm^2) in P10 control and *Rbpj*^{iPC} brain cortices. p-values, Student's t-test, n=4.
e, Transmission electron micrographs showing neurovascular units in the P10 *Rbpj*^{iPC} brain cortex. Note oedema formation (asterisks) around capillaries (top and centre). The centre panel shows an extreme case of collapsed vessel lumen. At early stages (P7, bottom), pericytes induce local constriction (arrowhead) of the vessel lumen. Scale bar, 5 μm (top and centre) or 500 nm (bottom).
f, **g**, Confocal images showing P10 brain cortices stained for IB4 (blood vessels and inflammatory cells of macrophage/monocyte lineage, red) and microtubule-associated protein 2 (MAP2, white) (**f**) or NeuN (green) and neurofilament-H (NF-H, white) (**g**). Areas where the neuronal-specific markers are lost (asterisks) are associated to the vascular defects and the strong secondary inflammatory response. Scale bar, 50 μm.
Error bars represent s.e.m. Source data are provided as a Source Data file.

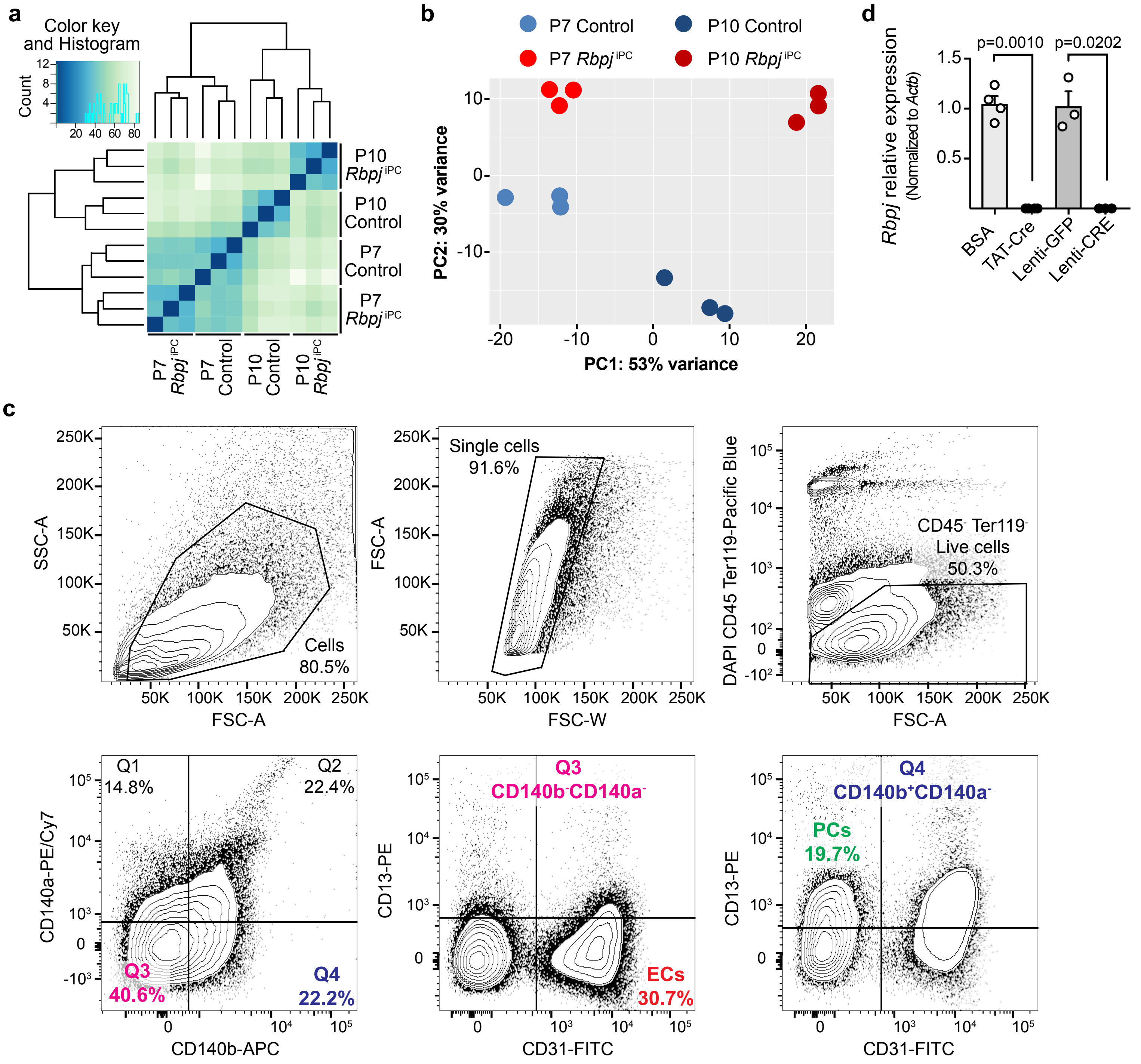

Supplementary Figure 6. RNA-seq, FACS and *in vitro* inactivation of *Rbpj*.
a, Unsupervised hierarchical clustering of RNA-seq datasets from P7 and P10 control and *Rbpj*^{iPC} mural cells.
b, Two-dimensional principal component analysis (PCA) plot illustrating the discrimination of gene expression profiles from control and *Rbpj*^{iPC} mural cells at different developmental stages.
c, Gating strategy for the isolation of ECs and mural cells from P7 and P10 brain cortices. After initial forward scatter (FSC)/side scatter (SSC) gating and exclusion of doublets, live cells negative for CD45 and Ter119 were gated and subdivided into PDGFRα$^-$ (CD140a) PDGFRβ$^-$ (CD140b) (Q3) from which the EC fraction (CD31$^+$, CD13$^-$) was sorted, and PDGFRα$^-$ PDGFRβ$^+$ (Q4) from which the mural cell fraction (CD13$^+$, CD31$^-$) was derived. This gating strategy was used for all FACS-derived data in the present manuscript.
d, Relative gene expression of *Rbpj* in cultured mouse brain primary pericytes by RT-qPCR after gene inactivation mediated by TAT-Cre or Lentivirus-Cre infection compared to controls (BSA-treated or Lentivirus-GFP infection, respectively). p-values, Welch's t-test, n=4 (BSA vs. TAT-Cre) or n=3 (Lenti-GFP vs. Lenti-Cre).
Error bars represent s.e.m. Source data are provided as a Source Data file.

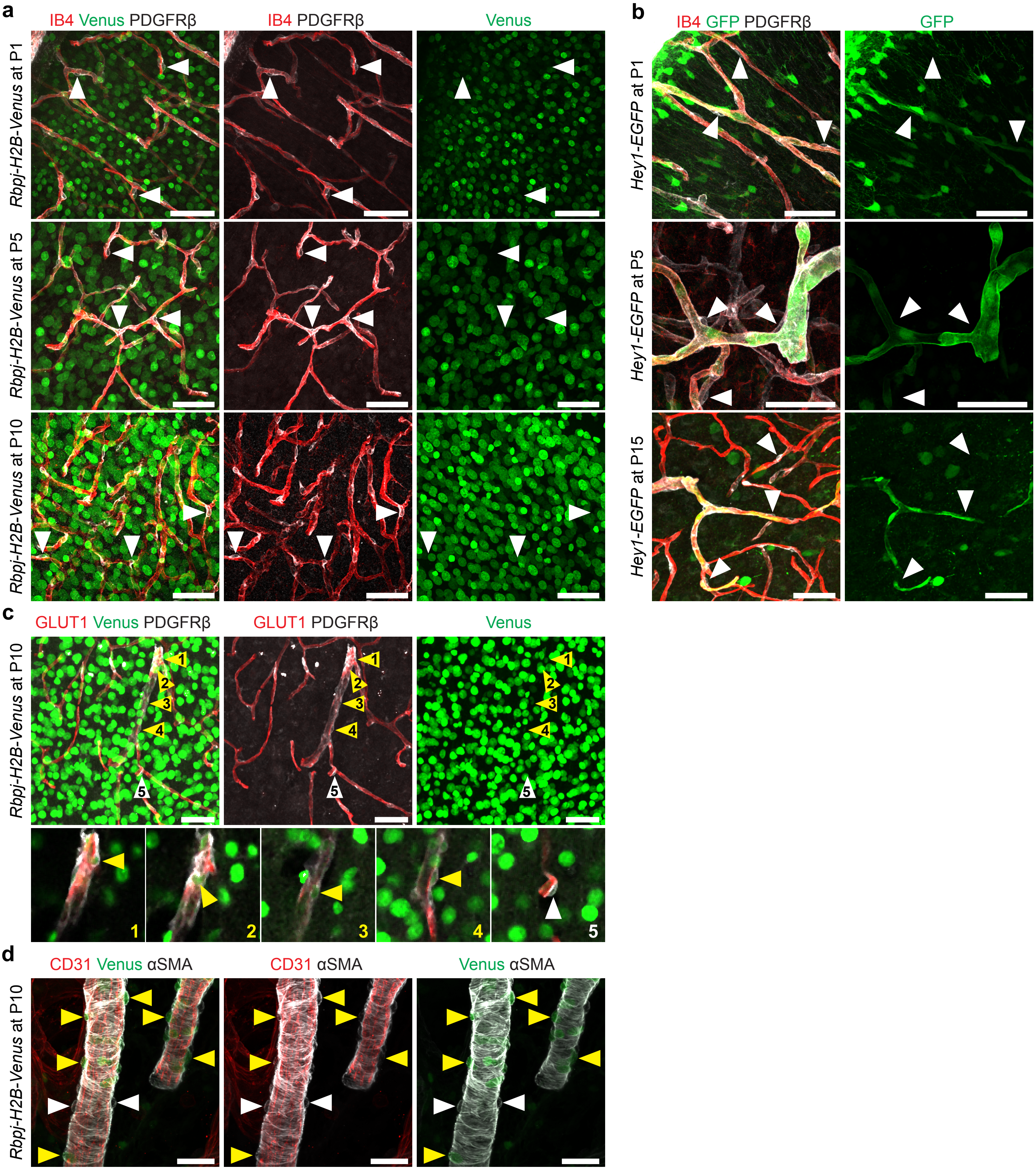

Supplementary Figure 7. Analysis of canonical Notch signalling in brain mural cells.
a, **b**, Confocal images showing absence of the Venus or EGFP reporter expression (**a**, and **b**, respectively, both in green) in PDGFRβ⁺ pericytes (white, arrowheads). Blood vessels are labelled by IB4 staining (red). Scale bar, 50 μm.
c, **d**, Confocal images showing frequent detection of nuclear Venus fluorescence (green) in PDGFRβ⁺ or αSMA⁺ (c, and d, respectively, both in white) SMCs associated with penetrating arterioles in the cortex (**c**), or pial arteries (**d**) in the surface of P10 mouse brains. Blood vessels are labelled with GLUT1 or CD31 (**c**, and **d**, respectively, both in red). Bottom panel in **c**, shows single optical sections of individual SMCs for better appreciation of the nuclear fluorescence signal. Numbers correspond to the highlighted SMCs, which are positive (yellow arrowheads) or negative (white arrowheads) for reporter signal. Scale bar, 50 μm in **c**, 25 μm in **d**.

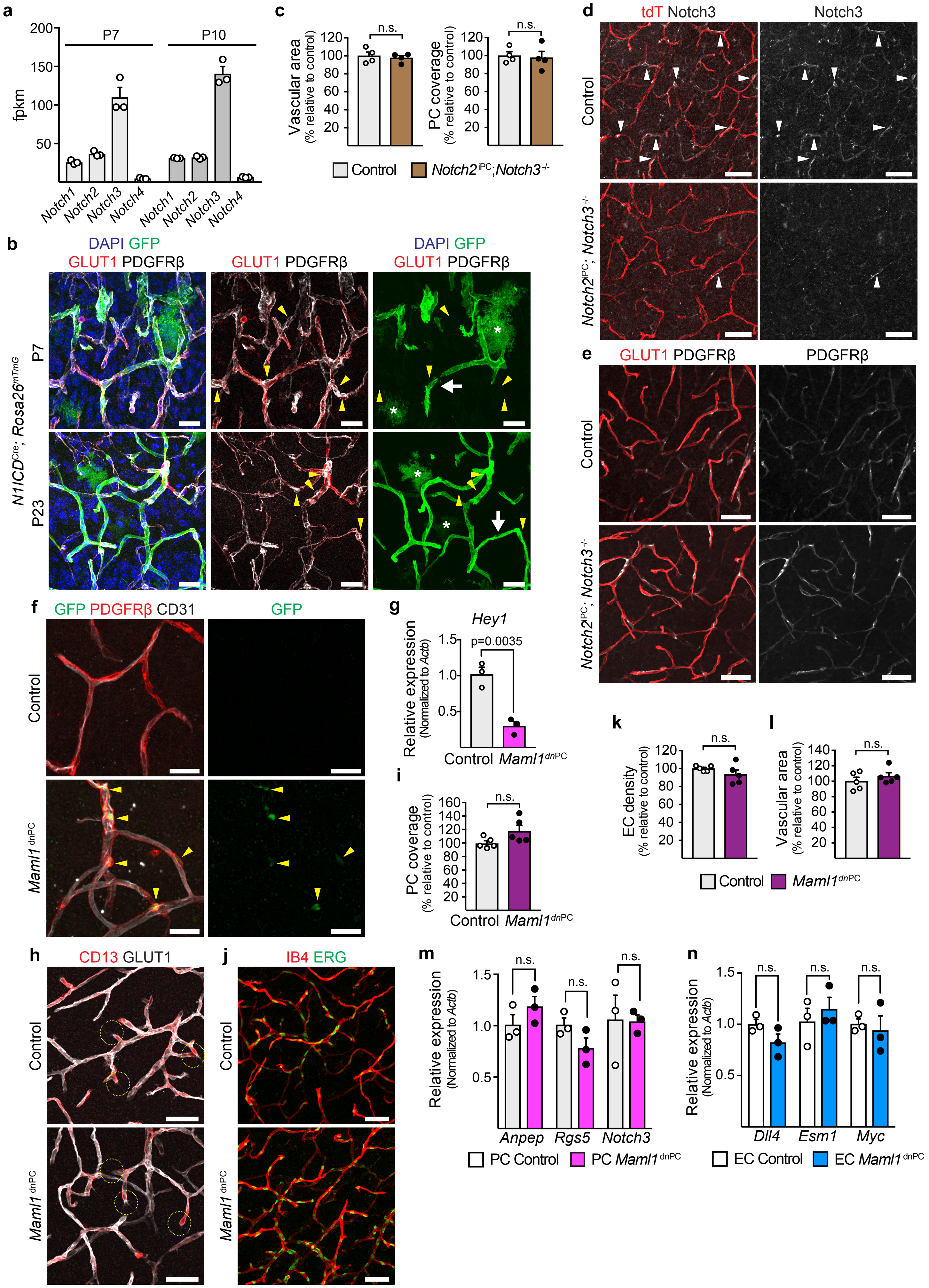

a
fpkm
P7
P10
Notch1
Notch2
Notch3
Notch4
b
DAPI GFP GLUT1 PDGFRβ
GLUT1 PDGFRβ
N1ICDCre; Rosa26mTmG
P7
P23
c
Vascular area (% relative to control)
PC coverage (% relative to control)
n.s.
Control
Notch2iPC;Notch3-/-
d
tdT Notch3
Notch3
Control
Notch2iPC; Notch3-/-
e
GLUT1 PDGFRβ
PDGFRβ
f
GFP PDGFRβ CD31
GFP
Maml1 dnPC
g
Hey1
p=0.0035
Relative expression (Normalized to Actb)
Control
Maml1dnPC
i
PC coverage (% relative to control)
k
EC density (% relative to control)
l
Vascular area (% relative to control)
h
CD13 GLUT1
j
IB4 ERG
m
Anpep
Rgs5
Notch3
PC Control
PC Maml1 dnPC
n
Dll4
Esm1
Myc
EC Control
EC Maml1 dnPC

Supplementary Figure 8. Notch signalling inhibition does not phenocopy *Rbpj* deletion.
a, Expression levels (fpkm) of Notch receptors in control brain cortex mural cells. n=3.
b, Confocal images showing GFP (green) expression after Cre-mediated recombination driven by *Notch1*$^{tm3(cre)Rko}$/J (*N1ICD*^{Cre}). Note that pericytes (PDGFRβ^{+}, white) do not express GFP (yellow arrowheads) in contrast to ECs (GLUT1^{+}, red; white arrows) or glial cells (asterisks). Scale bar, 25 µm.
c, Vascular area and pericyte coverage in control and *Notch2*^{iPC} *Notch3*$^{-/-}$ P10 mice. p-values, Student's t-test, n=4. n.s., not statistically significant.
d, Confocal images of blood vessels (tdT^{+}, red) stained for Notch3 (white) in P10 brain cortex samples. Note almost complete absence of Notch3 staining in *Notch2*^{iPC} *Notch3*$^{-/-}$ pericytes (arrowheads). Scale bar, 50 µm.
e, Confocal images showing normal investment of pericytes (PDGFRβ^{+}, white) in *Notch2*^{iPC} *Notch3*$^{-/-}$ P10 cortical vasculature (GLUT1^{+}, red). Scale bar, 50 µm.
f, Confocal images of cortical microvasculature (CD31, white) showing GFP (green) expression in recombined pericytes (PDGFRβ^{+}, red) of P10 *Maml1*^{dnPC} mice (yellow arrowheads). Scale bar, 25 µm.
g, RT-qPCR analysis of *Hey1* expression in sorted mural cells from P10 brain cortex. p-values, Student's t-test, n=3.
h, Confocal images showing normal sprouts formation (dashed circles) in P10 *Maml1*^{dnPC} mice. GLUT1 (white) and CD13 (red) reveal blood vessels and mural cells, respectively. Scale bar, 50 µm.
i, Quantitation of pericyte coverage in P10 control and *Maml1*^{dnPC} brain cortex vasculature. p-values, Student's t-test, n=5. n.s., not statistically significant.
j, Confocal images of cortical vasculature (IB4, red; ERG, green) showing normal EC density in P10 *Maml1*^{dnPC} mice. Scale bar, 50 µm.
k, **l**, EC density (**k**) and vascular area (**l**) in P10 control and *Maml1*^{dnPC} brain cortices. p-values, Welch's (**k**) or Student's (**l**) t-test, n=5. n.s., not statistically significant.
m, RT-qPCR analysis in freshly sorted mural cells from P10 control and *Maml1*^{dnPC} brain cortex. p-values, one-way ANOVA with Sidak's test, n=3. n.s., not statistically significant.
n, RT-qPCR analysis in freshly sorted ECs from P10 control and *Maml1*^{dnPC} brain cortex. p-values, Kruskal-Wallis with Dunn's test, n=3. n.s., not statistically significant.
Error bars represent s.e.m. Source data are provided as a Source Data file.

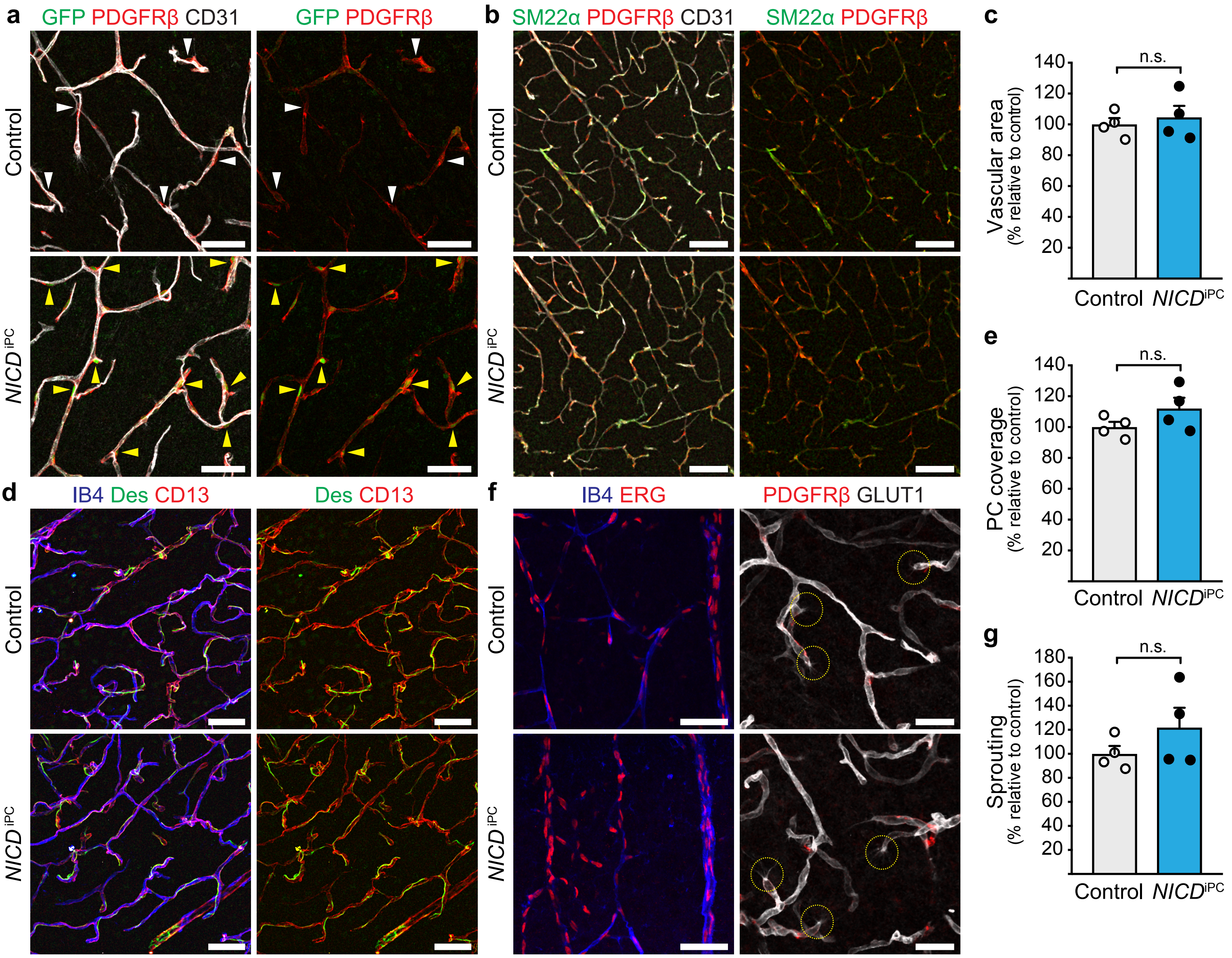

Supplementary Figure 9. Normal brain vascular morphogenesis in a Notch-gain of function model.

a, Confocal images of P10 control and *NICD*^{iPC} brain cortex vasculature (CD31^{+}, white). Expression of GFP (green, yellow arrowheads) marks recombined pericytes (PDGFRβ^{+}, red) after Cre-mediated activation of the *Rosa26-NICD* gain-of-function allele. Cre-negative littermates (control) show no GFP signal in mural cells (white arrowheads). Scale bar, 50 μm.

b, Confocal images showing comparable expression of the markers SM22α (green) and PDGFRβ (red) in the P10 *NICD*^{iPC} and control mouse brain cortex vasculature (CD31^{+}, white). Scale bar, 100 μm.

c, Vascular area in the brain cortices of control and *NICD*^{iPC} mice at P10. p-values, Student's t-test, n=4. n.s., not statistically significant.

d, Confocal images showing comparable expression of the pericyte markers Desmin (Des, green) and CD13 (red) in mural cells around blood vessels (IB4^{+}, blue) in P10 control and *NICD*^{iPC} brain cortices. Scale bar, 50 μm.

e, PC coverage in the brain cortices of control and *NICD*^{iPC} mice at P10. p-values, Student's t-test, n=4. n.s., not statistically significant.

f, Confocal images of the mouse brain cortex vasculature showing comparable density of EC nuclei (ERG, red) in P10 control and *NICD*^{iPC} blood vessels (IB4, blue). Likewise, GLUT1 staining (white), indicates normal sprouting (dashed circles). Scale bar, 50 μm for left panels and 100 μm for panels on the right.

g, Quantitation of sprouting in P10 control and *NICD*^{iPC} brain cortices. p-values, Student's t-test, n=4. n.s., not statistically significant.

Error bars represent s.e.m. Source data are provided as a Source Data file.

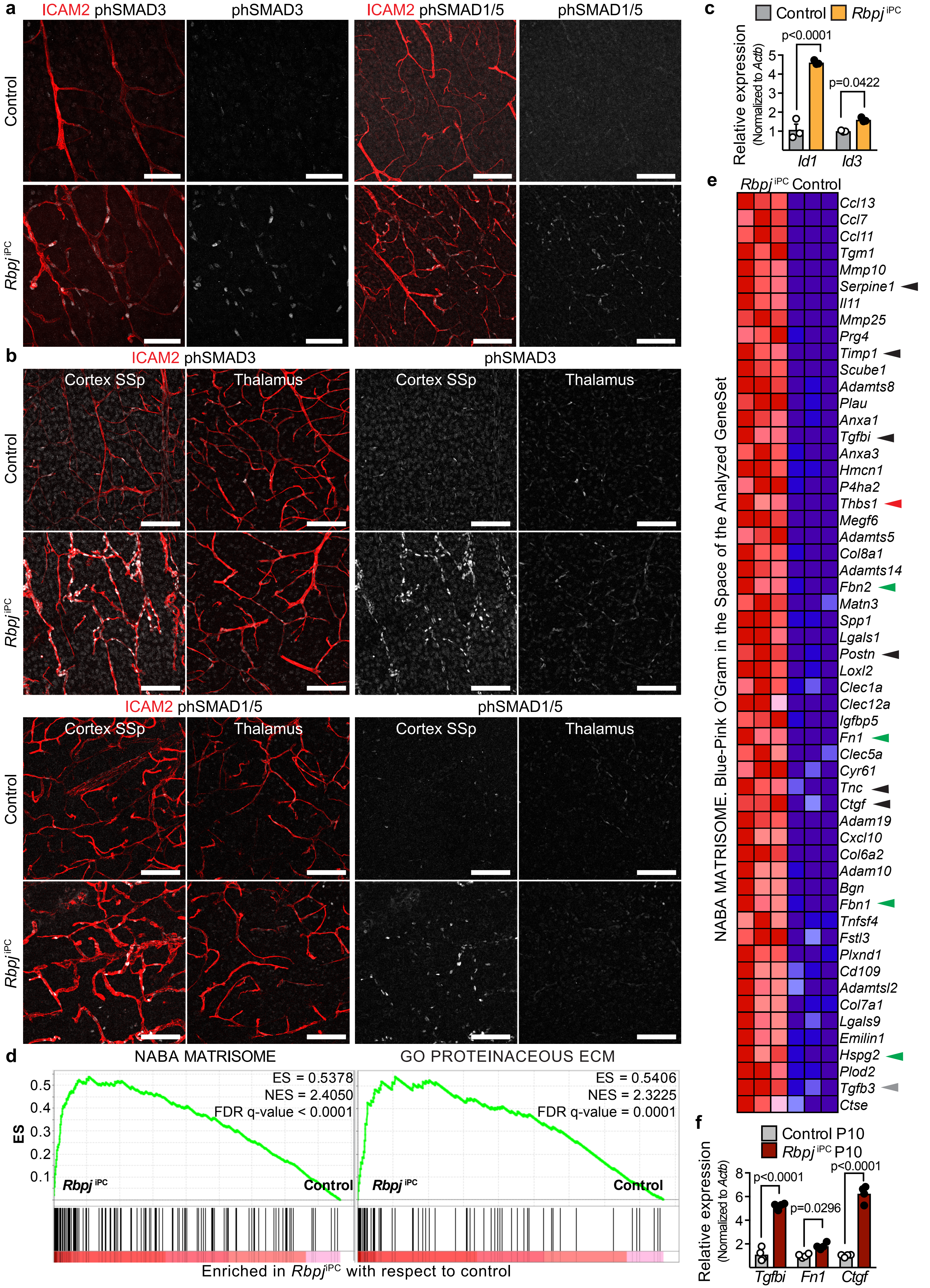
a
ICAM2 phSMAD3
phSMAD3
ICAM2 phSMAD1/5
phSMAD1/5
Control
Rbpj iPC
b
ICAM2 phSMAD3
phSMAD3
Cortex SSp
Thalamus
ICAM2 phSMAD1/5
phSMAD1/5
c
Control
Rbpj iPC
Relative expression (Normalized to Actb)
p<0.0001
p=0.0422
Id1
Id3
e
NABA MATRISOME. Blue-Pink O'Gram in the Space of the Analyzed GeneSet
Ccl13
Ccl7
Ccl11
Tgm1
Mmp10
Serpine1
Il11
Mmp25
Prg4
Timp1
Scube1
Adamts8
Plau
Anxa1
Tgfbi
Anxa3
Hmcn1
P4ha2
Thbs1
Megf6
Adamts5
Col8a1
Adamts14
Fbn2
Matn3
Spp1
Lgals1
Postn
Loxl2
Clec1a
Clec12a
Igfbp5
Fn1
Clec5a
Cyr61
Tnc
Ctgf
Adam19
Cxcl10
Col6a2
Adam10
Bgn
Fbn1
Tnfsf4
Fstl3
Plxnd1
Cd109
Adamtsl2
Col7a1
Lgals9
Emilin1
Hspg2
Plod2
Tgfb3
Ctse
d
NABA MATRISOME
GO PROTEINACEOUS ECM
ES
ES = 0.5378
NES = 2.4050
FDR q-value < 0.0001
ES = 0.5406
NES = 2.3225
FDR q-value = 0.0001
Rbpj iPC
Control
Enriched in Rbpj iPC with respect to control
f
Control P10
Rbpj iPC P10
Relative expression (Normalized to Actb)
p<0.0001
p=0.0296
p<0.0001
Tgfbi
Fn1
Ctgf

Supplementary Figure 10. Increased TGFβ signalling in the *Rbpj*^{iPC} cerebral vasculature.
a, Confocal images of the P7 *Rbpj*^{iPC} brain cortex vasculature showing increased phosphorylation of SMAD3 (phSMAD3, white, left panel) or SMAD1/5 (phSMAD1/5, white, right panel) in ICAM2^{+} blood vessels (red) relative to control. Note that vascular morphology in the mutants is not severely affected suggesting that increased phosphorylation of SMADs is not a secondary consequence in vascular lesions. Scale bar, 50 (left panel) and 100 µm (right panel).
b, Confocal images of the P10 *Rbpj*^{iPC} brain cortex vasculature showing increased phosphorylation of SMAD3 and SMAD1/5 (white) in ICAM2^{+} blood vessels (red) relative to littermate control. Note that in the thalamus, where vascular lesions are absent, the immunoreactivity for phosphorylated SMADs is not changed. Scale bar, 100 µm.
c, RT-qPCR analysis of BMP pathway target genes in sorted ECs from P7 control and *Rbpj*^{iPC} brain cortices. p-values, one-way ANOVA with Sidak's multiple comparison test, n=3.
d, GSEA of consistently upregulated transcripts from RNA-seq model-based clustering analysis showing enrichment of genes associated to the extracellular matrix in *Rbpj*^{iPC} mutants relative to control. ES, enrichment score; NES, normalized enrichment score; FDR, false discovery rate.
e, Heat map of the top 55 enriched genes from the Naba Matrisome signature (ensemble of genes encoding ECM and ECM-associated proteins) in *Rbpj*^{iPC} mural cells compared to control. Arrowheads indicate ECM proteins induced by TGFβ (black), basement membrane components involved in TGFβ sequestration and concentration (green), TGFβ-activators (red) and overexpressed TGFβ ligand (grey).
f, RT-qPCR analysis of ECM proteins known to be upregulated by TGFβ in sorted mural cells from control and *Rbpj*^{iPC} brains. p-values, one-way ANOVA with Sidak's test, n=4.
Error bars represent s.e.m. Source data are provided as a Source Data file.

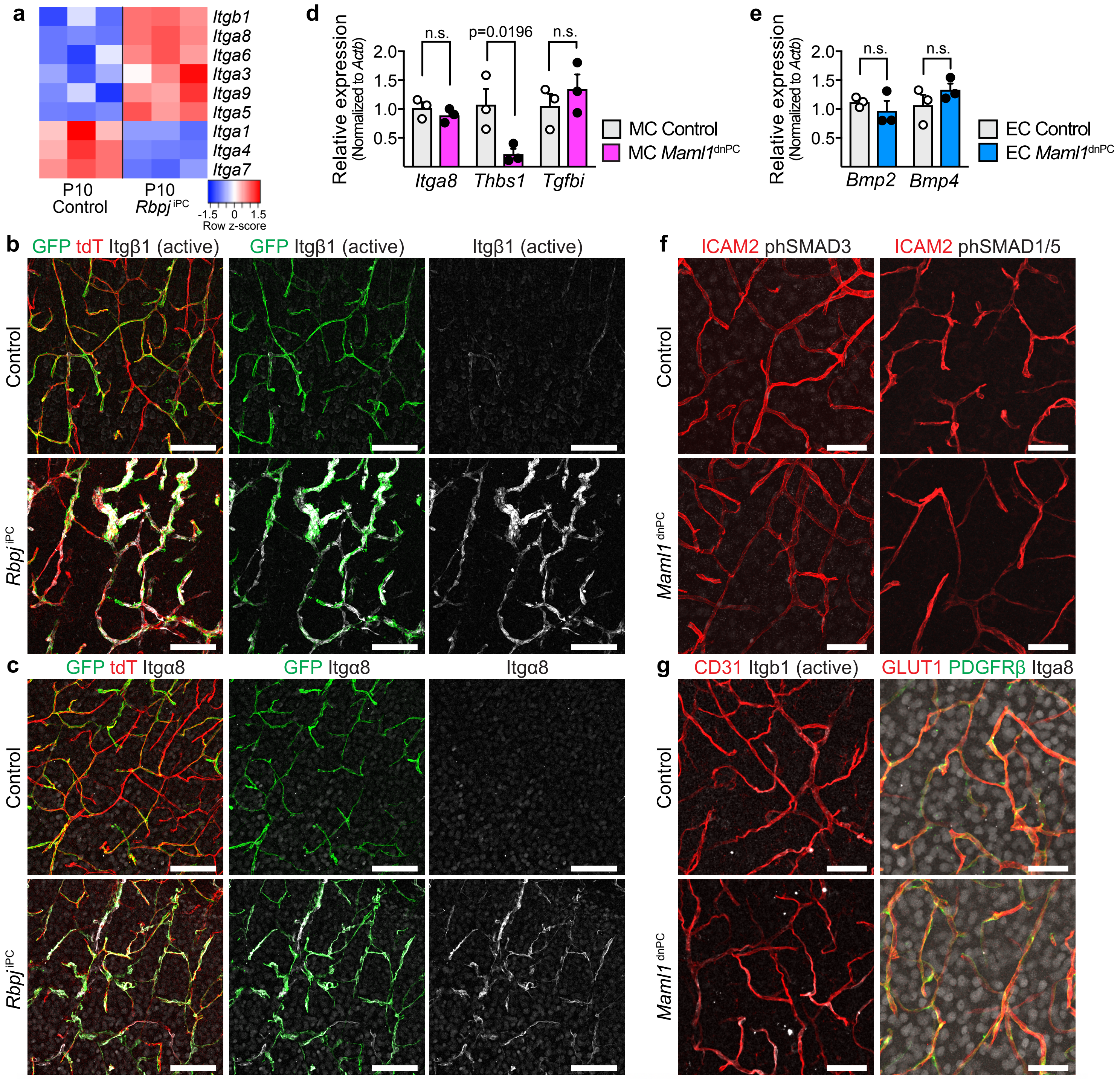

Supplementary Figure 11. Changes in ECM-cellular crosstalk in *Rbpj*^{iPC} mutants.

a, Heat map representation of integrin subunits showing changes in their expression pattern in P10 *Rbpj*^{iPC} brain mural cells relative to controls.

b, Confocal images showing increased activation of integrin β1 (Itgβ1, white) in the P10 *Rbpj*^{iPC} vasculature. Recombined pericytes (GFP^{+}, green) are present in close proximity to the endothelium (tdT^{+}, red). Scale bar, 100 µm.

c, Confocal images showing increased expression of integrin α8 (Itgα8, white) in P10 *Rbpj*^{iPC} pericytes (GFP^{+}, green). Blood vessels are labelled by tdT expression (red). Scale bar, 100 µm.

d, RT-qPCR showing that *Itga8*, *Thbs1* and *Tgfbi*, which are overexpressed in *Rbpj*^{iPC} mural cells, are not significantly altered in freshly sorted mural cells from P10 *Maml1*^{dnPC} brain cortex. p-values, one-way ANOVA with Sidak's test, n=3. n.s., not statistically significant.

e, RT-qPCR analysis showing that *Bmp2* and *Bmp4*, which are overexpressed in the *Rbpj*^{iPC} endothelium, are not significantly altered in the freshly sorted ECs from P10 *Maml1*^{dnPC} brain cortex. p-values, Kruskal-Wallis with Dunn's test, n=3. n.s., not statistically significant.

f, Confocal images showing normal levels of SMAD3 and SMAD1/5 phosphorylation (phSMAD3 and phSMAD1/5, white) in ICAM2-stained (red) blood vessels of the P10 control and *Maml1*^{dnPC} brain cortex. Scale bar, 50 µm.

g, Confocal images of P10 control and *Maml1*^{dnPC} brain cortex vasculature stained with CD31 (red) or GLUT1/PDGFRβ (red/green) showing no differences in the activation of integrin β1 (Itgβ1, white) or in the expression of integrin α8 (Itgα8, white). Scale bar, 50 µm.

Error bars represent s.e.m. Source data are provided as a Source Data file.

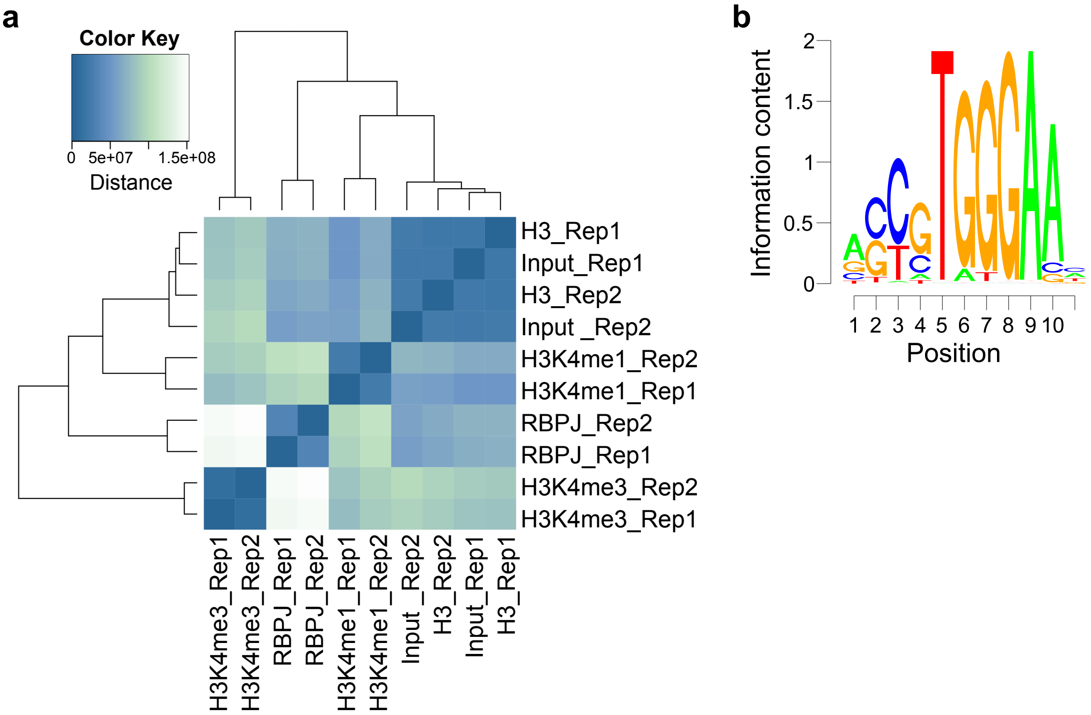

c

Motif logos		Motif source	ALT ID	Consensus	Width	Sites	E-value	Most similar motif
		DREME	DREME-1	TTCYCAB	7	3164	1.7e-414	MA1116.1 (RBPJ)
		MEME	MEME-1	NBYSTGGGAAM	11	3213	4.0e-135	MA1116.1 (RBPJ)
		DREME	DREME-2	CYCCDCCC	8	1928	2.9e-92	MA0079.3 (SP1)

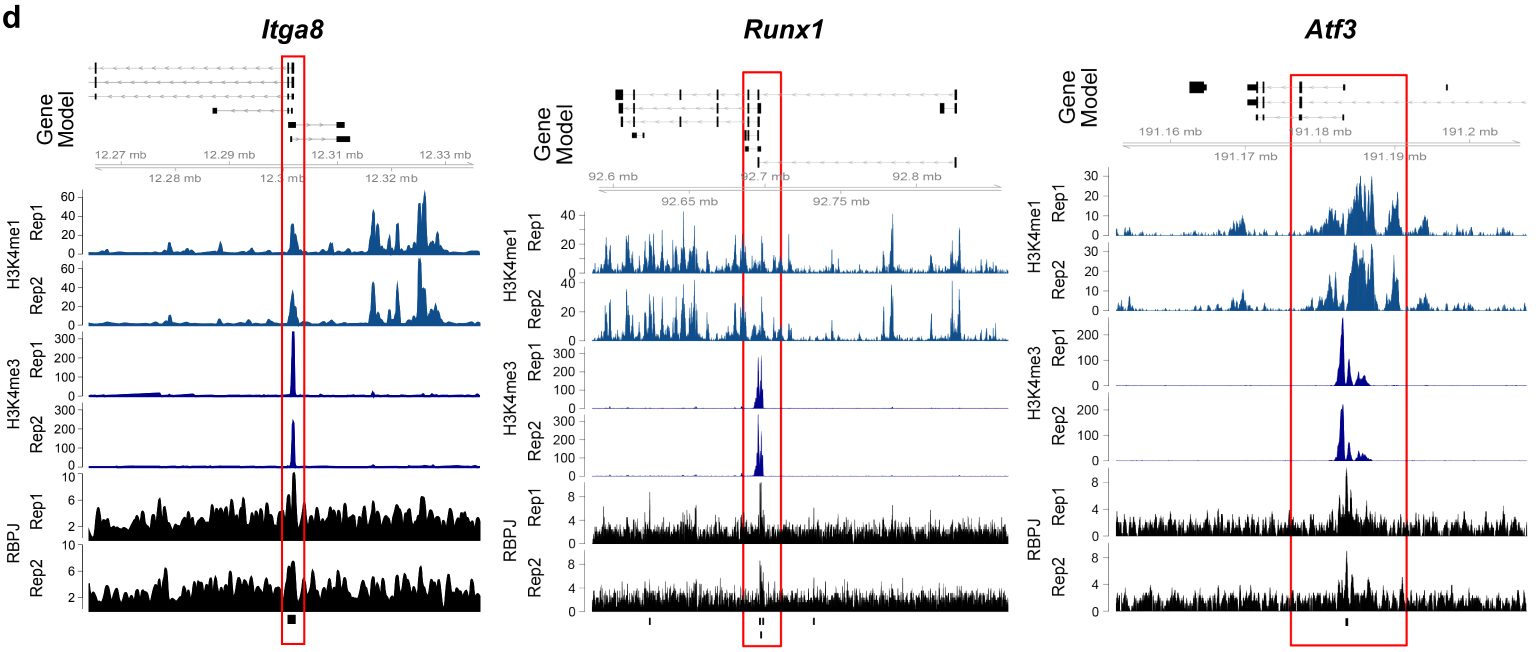

Supplementary Figure 12. Analysis of the genomic landscape associated to RBPJ binding in cultured pericytes.

a, Clustering analysis of distances ChIP-seq binding data collected across 1-Kb bins identifies experimental group memberships as expected and shows consistency across all data sets.

b, rGADEM motif analysis, performed on RBPJ ChIP-seq data in pericytes identifies the RBPJ binding motif.

c, MEME and DREME analysis, performed on RBPJ ChIP-seq data in pericytes, identifies the RBPJ binding motif amongst the top ranked motifs.

d, Snapshots showing the RBPJ binding along with the distribution of H3K4me1 and H3K4me3 at the *Itga8*, *Runx1* and *Atf3* loci in pericytes. Black bars below RBPJ profiles indicate the position of peak intervals. Two replicates (Rep1 and Rep2) of each experiment are shown.

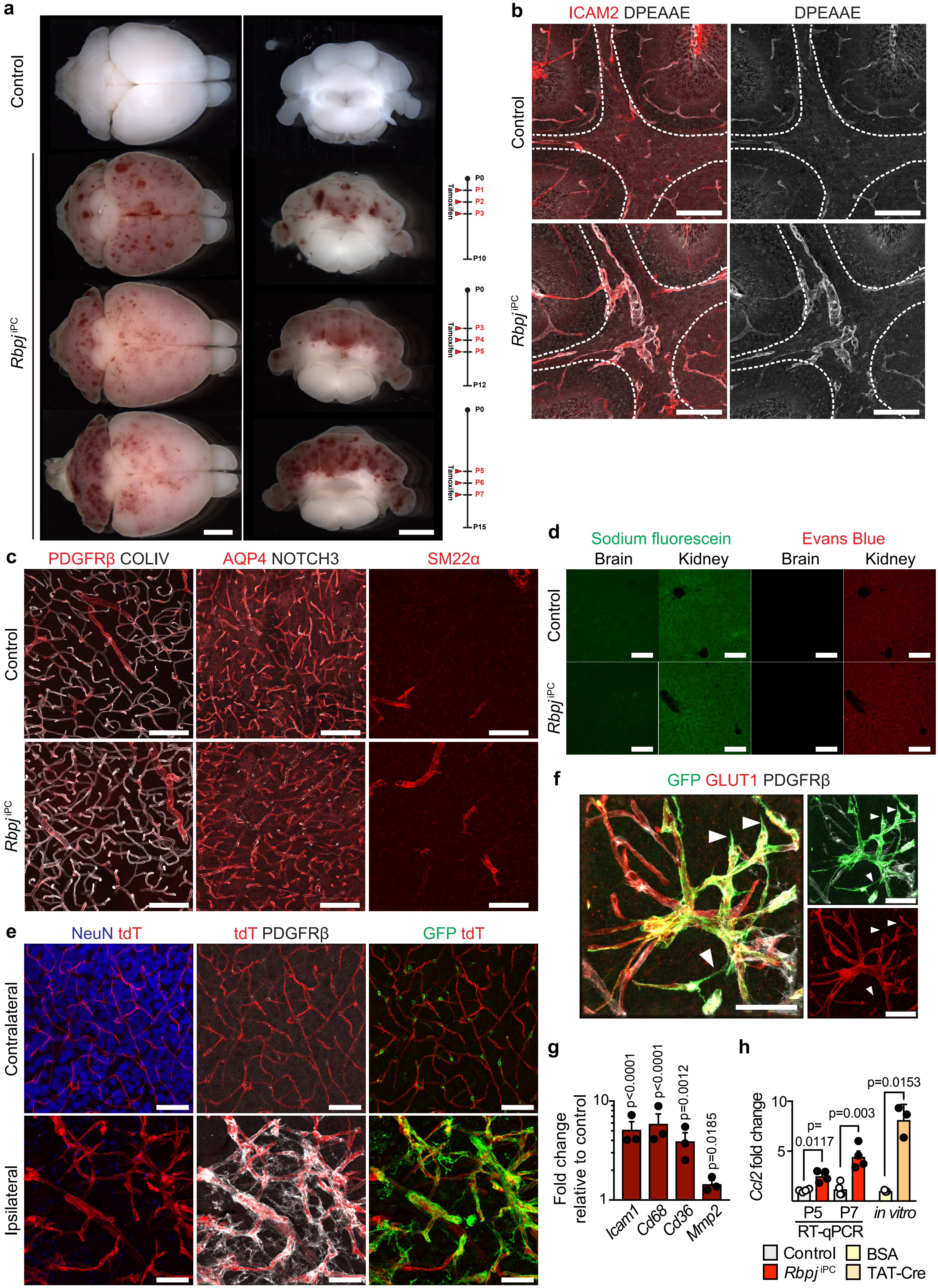

a
Control
Rbpj iPC
Tamoxifen
P0
P1
P2
P3
P10
P4
P5
P12
P6
P7
P15
b
ICAM2 DPEAAE
DPEAAE
Control
Rbpj iPC
c
PDGFRβ COLIV
AQP4 NOTCH3
SM22α
Control
Rbpj iPC
d
Sodium fluorescein
Evans Blue
Brain
Kidney
Brain
Kidney
Control
Rbpj iPC
e
NeuN tdT
tdT PDGFRβ
GFP tdT
Contralateral
Ipsilateral
f
GFP GLUT1 PDGFRβ
g
Fold change relative to control
p<0.0001
p<0.0001
p=0.0012
p=0.0185
10
1
Icam1
Cd68
Cd36
Mmp2
h
Ccl2 fold change
p=0.0153
p=0.003
p= 0.0117
10
5
P5
P7
in vitro
RT-qPCR
Control
Rbpj iPC
BSA
TAT-Cre

Supplementary Figure 13. Pathological consequences of *Rbpj* deletion in mural cells.
a, Macroscopic images from control and *Rbpj*^{iPC} brains at different developmental stages. Note gradual increase in the severity of the cerebellar lesions upon later induction of recombination. Tamoxifen administration regimes are indicated on the right. Scale bar, 2 mm.
b, Confocal images showing cleaved Versican (DPEAAE, white) in close proximity to blood vessels (ICAM2$^+$, red) in the *Rbpj*^{iPC} cerebellar white matter (dashed line). Scale bar, 100 μm.
c, Confocal images from the brain cortex vasculature of 8 months old control and *Rbpj*^{iPC} mice after tamoxifen administration at 3 weeks of age. Note normal mural cell (PDGFRβ$^+$, red, left) coverage, marker expression (NOTCH3, white, centre and SM22α, red, right), collagen IV (COLIV, white, left) deposition and astrocyte endfeet (AQP4$^+$, red, centre) polarization. Scale bar, 100 μm.
d, Confocal images of brain and kidney sections from 8 months old mice injected with sodium fluorescein and Evans Blue to assess BBB integrity in *Rbpj*^{iPC} mice. Scale bar, 100 μm.
e, Confocal images showing the infarct zone (ipsilateral) and a comparable region of the contralateral brain hemisphere from *Pdgfrb-CreERT2 Rosa26*^{mTmG} *Rbpj*$^{+/lox}$ mice subjected to dMCAO. Note strong reduction in neuronal marker expression (NeuN$^+$, blue, left), drastic increase in PDGFRβ (white, centre) and substantial changes in vessel (tdT$^+$, red) and pericyte (GFP$^+$, green, right) morphology after stroke. Scale bar, 50 μm.
f, Confocal images showing pericyte projections (GFP$^+$, green; PDGFRβ$^+$, white; arrowheads) emerging from blood vessels (GLUT1$^+$, red) in the infarct zone. Scale bar, 50 μm.
g, Gene expression fold change (based on RNA-seq counts) of inflammation-associated adhesion molecules (*Icam1*), phagocytic/endocytic receptors (*Cd68*, *Cd36*) and matrix metalloproteinases (*Mmp2*) in P10 *Rbpj*^{iPC} mural cells compared to controls (set as 1). n=3. p-adjusted values.
h, Gene expression analysis of chemokine *Ccl2* in sorted mural cells from control and *Rbpj*^{iPC} mice at P5 and P7, or cultured pericytes (BSA vs. TAT-Cre). p-values, Welch's or Student's t-test (RT-qPCR at P5 or P7, respectively), n=4 or Welch's t-test (*in vitro*), n=3.
Error bars represent s.e.m. Source data are provided as a Source Data file.

Experiment name	Total	Mapped	Properly paired	% properly paired
Input_Rep1	80445228	52889794	52889794	65.74634
Input_Rep2	102601590	66593678	66593678	64.90511
H3_Rep1	73546238	50534764	50534764	68.71156
H3_Rep2	86185816	59377732	59377732	68.89502
H3K4me1_Rep1	72065206	52457996	52457996	72.7924
H3K4me1_Rep2	93401102	66689756	66689756	71.40147
H3K4me3_Rep1	72330636	50660912	50660912	70.04074
H3K4me3_Rep2	76183514	55276612	55276612	72.55718
RBPJ_Rep1	225571982	124414852	124414852	55.15528
RBPJ_Rep2	230809950	128260744	128260744	55.56985

Supplementary Table 1. Mapping statistics of bowtie aligned reads to an index based on *GRCm38.92 (Ensembl)* version of the mouse genome.
