# Supplementary material for: Loss of the transcription factor RBPJ induces disease-promoting properties in brain pericytes
Source: Nat Commun. 2019 Jun 27;10:2817. doi: 10.1038/s41467-019-10643-w (PMC6597568; doi:10.1038/s41467-019-10643-w)
Supplement: Supplementary file 8 — Reporting Summary [file 41467_2019_10643_MOESM8_ESM.pdf]

nature research

Corresponding author(s): Ralf H. Adams
Peter Vajcokzy

Last updated by author(s): Apr 18, 2019

# Reporting Summary

Nature Research wishes to improve the reproducibility of the work that we publish. This form provides structure for consistency and transparency in reporting. For further information on Nature Research policies, see Authors & Referees and the Editorial Policy Checklist.

## Statistics

For all statistical analyses, confirm that the following items are present in the figure legend, table legend, main text, or Methods section.

| n/a | Confirmed | |
|---|---|---|
| ☐ | ☒ | The exact sample size (*n*) for each experimental group/condition, given as a discrete number and unit of measurement |
| ☐ | ☒ | A statement on whether measurements were taken from distinct samples or whether the same sample was measured repeatedly |
| ☐ | ☒ | The statistical test(s) used AND whether they are one- or two-sided<br>*Only common tests should be described solely by name; describe more complex techniques in the Methods section.* |
| ☒ | ☐ | A description of all covariates tested |
| ☐ | ☒ | A description of any assumptions or corrections, such as tests of normality and adjustment for multiple comparisons |
| ☐ | ☒ | A full description of the statistical parameters including central tendency (e.g. means) or other basic estimates (e.g. regression coefficient) AND variation (e.g. standard deviation) or associated estimates of uncertainty (e.g. confidence intervals) |
| ☐ | ☒ | For null hypothesis testing, the test statistic (e.g. *F*, *t*, *r*) with confidence intervals, effect sizes, degrees of freedom and *P* value noted<br>*Give P values as exact values whenever suitable.* |
| ☒ | ☐ | For Bayesian analysis, information on the choice of priors and Markov chain Monte Carlo settings |
| ☒ | ☐ | For hierarchical and complex designs, identification of the appropriate level for tests and full reporting of outcomes |
| ☒ | ☐ | Estimates of effect sizes (e.g. Cohen's *d*, Pearson's *r*), indicating how they were calculated |

*Our web collection on statistics for biologists contains articles on many of the points above.*

## Software and code

Policy information about availability of computer code

| | |
|---|---|
| Data collection | Volocity 6.3, ZEN Software 2.3 SP1 FP1 14.0, Leica Application Suite X 2.0.1.14392, FACS Diva 6.0, ImSpector Pro 5.1.347 |
| Data analysis | Volocity 6.3, ImSpector Pro 5.1.347, Photoshop CS6 13.0, Illustrator CS6 16.0.0, Fiji-IJ 2.0.0-rc-43, GraphPad Prism 7.0b. RNA-Seq data were analyzed using open source code TopHat2 2.1.1, HTSeq-count 0.6.1 and the packages DESeq2, TCC and MBClusterSeq.Gene symbols were annotated using biomaRt (BioConductor 3.1). Gene ontology analysis was performed using Advaita Bio's iPathwayGuide 1.4.0. ChIP-Seq data were analyzed using open source code fastqc 0.11.8, bowtie 1.1.2, MarkDuplicates (Picard), Samtoolsrmdup, bamCoverage (Deeptools) and the R/BioConductor package Gviz. Peak calling was done using Macs2 and Peakranger and identification of overrepresented functional terms was done with GREAT. computeMatrix and PlotHeatmap (Deeptools) were used for scores calculations per genome regions and heatmap representation. Motif identification was done using MEME-ChIP and rGADEM. Manipulation of sequencing reads was performed with Rsamtools and genomic intervals were represented as GenomicRanges objects. |

For manuscripts utilizing custom algorithms or software that are central to the research but not yet described in published literature, software must be made available to editors/reviewers. We strongly encourage code deposition in a community repository (e.g. GitHub). See the Nature Research guidelines for submitting code & software for further information.

## Data

Policy information about availability of data

All manuscripts must include a data availability statement. This statement should provide the following information, where applicable:

- Accession codes, unique identifiers, or web links for publicly available datasets
- A list of figures that have associated raw data
- A description of any restrictions on data availability

All relevant data supporting the results of the present study are included within the article, its Supplementary Information files and can be obtained from the corresponding author on reasonable request. The original RNA-Seq and ChIP-Seq data is available from the Gene Expression Omnibus (GEO) database under accession codes GSE117083 and GSE120482, respectively. A Source Data File is included which contains the raw data underlying the reported averages in all Figures

and Supplementary Figures.
List of Figures that have associated raw data in the Source Data file:
Figure 1c, d, e, g, j, l, n
Figure 2d, f, h
Figure 3c, e, f, g, h
Figure 4b, g, j, k
Figure 5a, c, e, h, i, j, k
Figure 6b
Figure 7b, d, e, h, k
Supplementary Figure 1c, f
Supplementary Figure 2b
Supplementary Figure 3c, d, j, k
Supplementary Figure 4c, d, e, h, i, j
Supplementary Figure 5b, d
Supplementary Figure 6d
Supplementary Figure 8a, c, g, i, k, l, m, n
Supplementary Figure 9c, e, g
Supplementary Figure 10c, d, e, f
Supplementary Figure 11a, d, e
Supplementary Figure 13g, h
Revision Figure 2a, b
Revision Figure 4a, b, c

# Field-specific reporting

Please select the one below that is the best fit for your research. If you are not sure, read the appropriate sections before making your selection.

☒ Life sciences ☐ Behavioural & social sciences ☐ Ecological, evolutionary & environmental sciences

For a reference copy of the document with all sections, see nature.com/documents/nr-reporting-summary-flat.pdf

# Life sciences study design

All studies must disclose on these points even when the disclosure is negative.

| | |
|---|---|
| Sample size | No statistical methods were used to predetermine sample size; instead sample number was defined according to previous experience and reproducibility of the results across several independent experiments. |
| Data exclusions | Data were only excluded for failed experiments, reasons for which included suboptimal gene recombination, mistakes in pipetting or calculation as revealed by obvious deviation in technical replicates. No outliers were identified by statistical testing. |
| Replication | Results are based on three or more independent experiments to guarantee reproducibility of the findings. For all in vivo experiments done the number of animals (n) analysed per group was derived from at least two different litters. The number of replicates is given in the respective figure legends. Individual values are shown in each figure. |
| Randomization | No randomization of mice was used. Randomization is not relevant to our study design. Mice analyzed were littermates and with equal distribution of males and females whenever possible. |
| Blinding | Investigators were not blinded to mouse genotypes during experiments. Data reported for mouse experiments are not subjective but rather based on quantitative analysis. In most instances phenotypic differences are obvious between control and mutant mice. |

# Reporting for specific materials, systems and methods

We require information from authors about some types of materials, experimental systems and methods used in many studies. Here, indicate whether each material, system or method listed is relevant to your study. If you are not sure if a list item applies to your research, read the appropriate section before selecting a response.

## Materials & experimental systems

| n/a | Involved in the study | |
|---|---|---|
| ☐ | ☒ | Antibodies |
| ☐ | ☒ | Eukaryotic cell lines |
| ☒ | ☐ | Palaeontology |
| ☐ | ☒ | Animals and other organisms |
| ☒ | ☐ | Human research participants |
| ☒ | ☐ | Clinical data |

## Methods

| n/a | Involved in the study | |
|---|---|---|
| ☐ | ☒ | ChIP-seq |
| ☐ | ☒ | Flow cytometry |
| ☐ | ☒ | MRI-based neuroimaging |

# Antibodies

| | |
|---|---|
| Antibodies used | All antibodies are commercially purchased and detailed information for them is provided in the Methods section. The following primary antibodies were used for immunostaining : rabbit anti-AQP4 (1:200, Sigma HPA014784), mouse anti-?SMA-Cy3 (1:300, Sigma C6198), mouse anti-?SMA-660 (1:300, eBioscience 50-9760-82), rat anti-CD13 (1:100, abD Serotec, MCA2183GA), goat anti CD31 (1:200, R&D Systems, AF3628), rat anti VE-cadherin (CDH5, 1:100, BD Biosciences, 555289), rabbit anti-Claudin 5 (CLDN5, 1:100, Thermo Fisher Scientific, 34-1600), rabbit anti-cleaved Caspase-3 (1:100, Cell Signaling, 9664), rabbit anti-Desmin (1:100, Abcam, ab15200), rabbit anti DPEAAE (1:100, Thermo Scientific, PA1-1748A), rabbit anti ERG1 (1:100, Abcam, ab110639), rabbit anti-GFAP (1:200, DAO, Z0334), chicken anti-GFP (1:300, 2BScientific Ltd., GFP-1010), rabbit anti-GLUT1 (1:200, Millipore, 07-1401), goat anti-Iba1 (1:100, Novus Biologicals, NB100-1028), rat anti-ICAM2 (1:200, BD Pharmingen, 553326), goat anti-integrin ?8 (Itg?8, 1:100, Thermo Fisher Scientific, FA5-47572), goat anti-KLF4 (1:100, R&D Systems, AF3158), rabbit anti MAP-2 (1:100, Abcam, ab32454), rat anti-nestin (1:200, Santa Cruz, sc101541), rabbit anti-NeuN (1:200, Cell Signalling, 12943), chicken anti-neurofilament H (NF-H, 1:100, Neuromics, CH22104), rabbit anti-NG2 (1:100, Millipore, AB5320), goat anti-Notch3 (1:100, R&D Systems, AF1308), rat anti-PDGFRβ (1:100, eBioscience, 14-1402), goat anti-PDGFR PDGFRβ (1:100, &D Systems, AF1042), rabbit anti-phospho myosin light chain 2 (phMLC2, 1:100, Cell Signalling, 3674), rabbit anti-phosphoSMAD3 (phSMAD3, 1:100, Abcam, ab52903), rabbit anti-phospho SMAD1/5 (phSMAD1/5, 1:100, Cell Signalling, 9516), goat anti-podocalyxin (PODXL, 1:200, R&D Systems, AF1556), rabbit anti SM22? (1:200, Abcam, ab14106), rat anti-TER-119 (1:200, R&D Systems, MAB1125), rabbit anti-thrombospondin 1 (Thbs1, 1:100, Abcam, ab85762), goat anti-VEGR2 (1:100, R&D, AF644), rabbit anti-vimentin (1:100, Abcam, ab7783) and rabbit anti-zona occludens 1 (ZO1, 1:100, Invitrogen, 402200). In addition, biotin-conjugated isolectin B4 (IB4, 1:25, Vector Laboratories, B-1205) was used for visualization of blood vessels and inflammatory cells.<br>The following donkey-raised secondary antibodies (all in 1:400 dilution unles otherwise stated) were used for immunostaining: anti-rabbit IgG conjugated to Alexa Fluor (AF) 488 (Thermo Fisher Scientific, A21206), anti-chicken IgY AF488 (Jackson ImmunoResearch, 703-545-155), anti-rat IgG AF488 (Thermo Fisher Scientific, A21208), anti goat IgG AF488 (Invitrogen, A-11055), anti-rat IgG Cy3 (Jackson ImmunoResearch, 712-165-153), anti-rabbit IgG AF546 (Thermo Fisher Scientific, A10040), anti-goat IgG AF546 (Invitrogen, A-11056), anti-rat IgG AF594 (Thermo Fisher, A21209), anti-rabbit IgG AF594 (Thermo Fisher Scientific, A21207), anti-goat IgG AF594 (Thermo Fisher Scientific, A-11058), anti-rabbit IgG AF647 (Thermo Fisher Scientific, A31573), anti-rat IgG AF647 (Jackson ImmunoResearch, 712-605-153) and anti-goat IgG AF647 (Thermo Fisher Scientific, A21447). Streptavidin AF405 (1:200, Invitrogen S32351) was used for detection of biotinilated-IB4 stained samples. |
| Validation | Primary antibodies were validated by the manufacturer and confirmed by specific labeling of target molecules or cell types. Secondary antibodies have been tested in our experimental conditions to rule out unspecific reactivity. |

# Eukaryotic cell lines

Policy information about cell lines

| | |
|---|---|
| Cell line source(s) | Mouse primary brain pericytes were isolated according to previously published protocols. A detailed description of the procedure is available in the Methods section (Primary brain pericytes isolation and culture). |
| Authentication | Expression of known pericyte markers was assessed by RT-qPCR and immunohistochemistry. |
| Mycoplasma contamination | Primary brain pericytes were not tested for mycoplasma contamination. |
| Commonly misidentified lines<br>(See ICLAC register) | No misidentified lines were used in this study |

# Animals and other organisms

Policy information about studies involving animals; ARRIVE guidelines recommended for reporting animal research

| | |
|---|---|
| Laboratory animals | A full list of the mouse strains used is detailed in the Methods Section. No other laboratory animals were used. Both male and female mice were used in all cases.<br>Pdgfrb(BAC)CreERT2. JAX 029684. Age of analysis: E14.5, P5, P6, P7, P10, P12, P15, juvenile (3 weeks), adult (8 and >20 weeks) and aged (>70 weeks).<br>Rbpj-lox. MGI:3583755. Age of analysis: P5, P7, P10, P12, P15, juvenile (3 weeks), adult (8 and >20 weeks).<br>Rosa26mTmG. JAX 007576. Age of analysis: E14.5, P7, P10, P15, juvenile (3 weeks), adult (8 and >20 weeks) and aged (>70 weeks).<br>Rpl22 (RiboTag). JAX 011029. Age of analysis: P7 and P10.<br>Rosa26-DTA. JAX 032087. Age of analysis: P6.<br>Pdgfb-ret. MGI:2670574. Age of analysis: P9.<br>Notch1-Cre. JAX 006953. Age of analysis: P7 and P23.<br>Notch3-KO. JAX 010547. Age of analysis: P10.<br>Notch2-lox. JAX 010525. Age of analysis: P10.<br>Rosa26-dnMaml1. MGI:5426507. Age of analysis: P10.<br>Rosa26-NICD. JAX 008159. Age of analysis: P10.<br>Hey1-EGFP. MGI:4847129. Age of analysis: P1, P5 and P15.<br>CBF:H2B-Venus. JAX 020942. Age of analysis: P1, P5 and P10. |
| Wild animals | No wild animals were used in this study. |

Field-collected samples

No field-collected samples were used in this study.

Ethics oversight

All experiments involving animals were performed in compliance with the relevant laws and institutional guidelines, following protocols previously approved by local animal ethics committees and conducted with permission granted by the Landesamt für Natur, Umwelt und Verbraucherschutz (LANUV) of North Rhine-Westfalia to the Max Planck Institute of Molecular Biomedicine and by the Landesamt für Gesundheit und Soziales (G0298/13) of Berlin to the Center for Stroke-Research Berlin (CSB), Charité-Universitätsmedizin.

Note that full information on the approval of the study protocol must also be provided in the manuscript.

# ChIP-seq

## Data deposition

☒ Confirm that both raw and final processed data have been deposited in a public database such as GEO.

☒ Confirm that you have deposited or provided access to graph files (e.g. BED files) for the called peaks.

Data access links
*May remain private before publication.*

https://www.ncbi.nlm.nih.gov/geo/query/acc.cgi?acc=GSE120482

Files in database submission

raw data:
RD1_10%_input_1I_Rep1.fastq.gz
RD1_10%_input_1I_Rep1_pair2.fastq.gz
RD1_10%_input_3I_Rep2.fastq.gz
RD1_10%_input_3I_Rep2_pair2.fastq.gz
RD1_H3_1D_Rep1.fastq.gz
RD1_H3_1D_Rep1_pair2.fastq.gz
RD1_H3_3D_Rep2.fastq.gz
RD1_H3_3D_Rep2_pair2.fastq.gz
RD1_H3K4me1_1B_Rep1.fastq.gz
RD1_H3K4me1_1B_Rep1_pair2.fastq.gz
RD1_H3K4me1_3B_Rep2.fastq.gz
RD1_H3K4me1_3B_Rep2_pair2.fastq.gz
RD1_H3K4me3_1C_Rep1.fastq.gz
RD1_H3K4me3_1C_Rep1_pair2.fastq.gz
RD1_H3K4me3_3C_Rep2.fastq.gz
RD1_H3K4me3_3C_Rep2_pair2.fastq.gz
RD1_RBPJ_1E_Rep1.fastq.gz
RD1_RBPJ_1E_Rep1_pair2.fastq.gz
RD1_RBPJ_3E_Rep2.fastq.gz
RD1_RBPJ_3E_Rep2_pair2.fastq.gz

coverage vectors:
RD1_10%_input_1I_Rep1.fastq_Ensembl_GRCm38.92_mouse_sorted.bam_min.bam.bigwig
RD1_10%_input_3I_Rep2.fastq_Ensembl_GRCm38.92_mouse_sorted.bam_min.bam.bigwig
RD1_H3_1D_Rep1.fastq_Ensembl_GRCm38.92_mouse_sorted.bam_min.bam.bigwig
RD1_H3_3D_Rep2.fastq_Ensembl_GRCm38.92_mouse_sorted.bam_min.bam.bigwig
RD1_H3K4me1_1B_Rep1.fastq_Ensembl_GRCm38.92_mouse_sorted.bam_min.bam.bigwig
RD1_H3K4me1_3B_Rep2.fastq_Ensembl_GRCm38.92_mouse_sorted.bam_min.bam.bigwig
RD1_H3K4me3_1C_Rep1.fastq_Ensembl_GRCm38.92_mouse_sorted.bam_min.bam.bigwig
RD1_H3K4me3_3C_Rep2.fastq_Ensembl_GRCm38.92_mouse_sorted.bam_min.bam.bigwig
RD1_RBPJ_1E_Rep1.fastq_Ensembl_GRCm38.92_mouse_sorted.bam_min.bam.bigwig
RD1_RBPJ_3E_Rep2.fastq_Ensembl_GRCm38.92_mouse_sorted.bam_min.bam.bigwig

Peaks for RBPJ:
RBPJ_peaks.bed

Genome browser session
(e.g. UCSC)

https://genome-euro.ucsc.edu/s/MarekB/mm10_RBPJ_NatCom

## Methodology

Replicates

Two biological replicates were performed for each target protein.

Replicates were compared on genome-wide scale with a binning scheme of consecutive 1-kb bins covering the complete Mus musculus GRC_m38.92 genome. The correlation coefficients (Pearson) were calculated for the pair-wise comparison of coverage vectors across these bins and were as follows:

RBPJ 0.9999698
Input: 0.9997533
H3: 0.9997938
H3K4me1: 0.9618874

| | |
|---|---|
| | H3K4me3: 0.9972927 |
| Sequencing depth | xperiment_name\|Total\|Mapped\|Properly_paired\|%_properly_paired<br>Input_Rep1\|80445228\|52889794\|52889794/65.7<br>Input_Rep2\|102601590\|66593678\|66593678\|64.9<br>H3_Rep1\|73546238\|50534764\|50534764\|68.7<br>H3_Rep2\|86185816\|59377732\|59377732\|68.8<br>H3K4me1_Rep1\|72065206\|52457996\|52457996\|72.7<br>H3K4me1_Rep2\|93401102\|66689756\|66689756\|71.4<br>H3K4me3_Rep1\|72330636\|50660912\|50660912\|70.0<br>H3K4me3_Rep2\|76183514\|55276612\|55276612\|72.5<br>RBPJ_Rep1\|225571982\|124414852\|124414852\|55.1<br>RBPJ_Rep2\|230809950\|128260744\|128260744\|55.5 |
| Antibodies | H3 (abcam, ab1791), H3K4me1 (abcam, ab8895), H3K4me3 (Diagenode, pAb-003-050) and RBPJ (Cell Signaling, #5313) |
| Peak calling parameters | macs2 callpeak -c /data4/Rodrigo_GEO/new_ENSEMBL/ENSEMBL_92/align/1/RD1_10%_input_1I_Rep1_pair2.fastq_Ensembl_GRCm38.92_mouse_sorted.bam_min.bam -t /data4/Rodrigo_GEO/new_ENSEMBL/ENSEMBL_92/align/9/RD1_RBPJ_1E_Rep1_pair2.fastq_Ensembl_GRCm38.92_mouse_sorted.bam_min.bam -g 2.2e+09 -n rbpj_peaks_mm10_macs2_macs2 -f BAMPE<br><br>macs2 callpeak -c /data4/Rodrigo_GEO/new_ENSEMBL/ENSEMBL_92/align/2/RD1_10%_input_3I_Rep2.fastq_Ensembl_GRCm38.92_mouse_sorted.bam_min.bam -t /data4/Rodrigo_GEO/new_ENSEMBL/ENSEMBL_92/align/10/RD1_RBPJ_3E_Rep2_pair2.fastq_Ensembl_GRCm38.92_mouse_sorted.bam_min.bam -g 2.2e+09 -n rbpj_peaks_2_mm10_macs2_macs2 -f BAMPE<br><br>peakranger ranger -c /data4/Rodrigo_GEO/new_ENSEMBL/ENSEMBL_92/align/1/RD1_10%_input_1I_Rep1_pair2.fastq_Ensembl_GRCm38.92_mouse_sorted.bam_min.bam -d /data4/Rodrigo_GEO/new_ENSEMBL/ENSEMBL_92/align/9/RD1_RBPJ_1E_Rep1_pair2.fastq_Ensembl_GRCm38.92_mouse_sorted.bam_min.bam --format=BAM -o rbpj_peaks_mm10_ranger_ranger -q 0.2 -t 4<br><br>peakranger ranger -c /data4/Rodrigo_GEO/new_ENSEMBL/ENSEMBL_92/align/2/RD1_10%_input_3I_Rep2.fastq_Ensembl_GRCm38.92_mouse_sorted.bam_min.bam -d /data4/Rodrigo_GEO/new_ENSEMBL/ENSEMBL_92/align/10/RD1_RBPJ_3E_Rep2_pair2.fastq_Ensembl_GRCm38.92_mouse_sorted.bam_min.bam --format=BAM -o rbpj_peaks_2_mm10_mranger_ranger -q 0.2 -t 4 |
| Data quality | All processing steps were quality controlled. Initial inspection of sequencing reads was done using FastQC. After mapping we used MultiQC for control of alignments. After extraction of FPKM normalized coverage vectors, all ChIP-seq data were visually inspected in genome browsers along with identified peak intervals in order to check for general consistency of the data. Furthermore, when available, we compared at this stage our data to publicly available data sets (for histone modifications) in order to validate the overall patterns observed in our data.  In order to select for high-confidence RBPJ binding sites, we called peaks from both replicates independently using two peak calling programs and selected those peaks being called in at least 3 of the 4 cases.<br><br>In case of peakranger we called 51774 and 67920 peaks respectively with FDR<0.05 and 5-fold enrichment of IP over input reads.<br><br>In case of macs2 we called 27737 and 27649 peaks respectively with FDR<0.05 and 3-fold enrichment of IP over input reads.<br><br>After stringent filtering applying above mentioned criteria we finally selected 11094 peaks. |
| Software | MultiQC 1.6; FastQC 0.11.5; Bowtie-1.1.2; samtools-1.3.1; picard-tools-2.1.1; deeptools 2.5.4.2;  macs2 2.1.1.2; peakranger 1.17; meme-chip 4.10.2; R 3.4.0; DESeq2 1.16; GenomicRanges 1.28.6; rGADEM 2.24; seqLogo 1.42; GREAT 3.0.0 |

# Flow Cytometry

## Plots

Confirm that:

☒ The axis labels state the marker and fluorochrome used (e.g. CD4-FITC).

☒ The axis scales are clearly visible. Include numbers along axes only for bottom left plot of group (a 'group' is an analysis of identical markers).

☒ All plots are contour plots with outliers or pseudocolor plots.

☒ A numerical value for number of cells or percentage (with statistics) is provided.

## Methodology

| | |
|---|---|
| Sample preparation | Brain cortices from P5, P7 or P10 pups were collected in ice cold dissection media (DMEM (Sigma, D6546) supplemented with penicillin/streptomycin (PAA, P11-010) and 25 mM HEPES (Sigma, H3537)) and transferred to digestion media (37°C pre-warmed dissection media supplemented with 100 µg mL-1 Liberase DH (Roche, 5401054) and 50 µg mL-1 DNAseI (Sigma, DN25). Samples were minced and digested for 15 minutes at 37°C with occasional pipetting to disaggregate tissue pieces. FACS buffer (PBS with |

| | |
|---|---|
| | 2% FCS) was added and the tissue homogenate was mixed with 1.7 volumes of 22% albumin fraction V (BSA, Carl Roth, 8076.2). After centrifugation (1000 g, 12 min at RT) the supernatant was removed and the cell pellet resuspended in 5 mL of FACS buffer, filtered through a 40 µm nylon mesh (Corning, 352340) and centrifuged (300 g, 5 min at RT). The cell pellet, consisting mostly of single cells was resuspended in a suitable volume of FACS buffer together with the antibodies used for staining which are described in detail in the Methods section (Fluorescence Assisted Cell Sorting (FACS) of brain pericytes and endothelial cells). |
| Instrument | Cell sorting was performed in a FACS Aria IIu (BD Biosciences) with a 70 µm nozzle. |
| Software | FACSDiva software (BD Bioscience, Version 6.0). |
| Cell population abundance | Purity of sorted fractions is assessed by gene expression analysis of putative markers for the specific cell population with respect to the input (single cell suspension before sorting) and the other cell fraction sorted (i.e. pericytes vs. endothelial cells). |
| Gating strategy | Exclusion of debris and doublets together with single cells selection was based on forward scatter area/side scatter area and forward scatter width/forward scatter area analysis. Live cells (DAPI-) that do not express CD45 and Ter119 were gated and subdivided into PDGFRα- PDGFRβ- (Q3) from where the EC fraction (CD31+, CD13-) is sorted, and PDGFRα- PDGFRβ+ (Q4) from where the pericyte fraction (CD13+, CD31-) is derived. Gating strategies to discriminate cell populations based on their immunolabelling were defined according to reference experiments using single-stained samples and FMO (fluorescence minus one) controls. A figure describing the gating strategy is shown in Supplementary Figure 6c. |

☒ Tick this box to confirm that a figure exemplifying the gating strategy is provided in the Supplementary Information.

# Magnetic resonance imaging

## Experimental design

| | |
|---|---|
| Design type | Stroke imaging - anatomical scan |
| Design specifications | N/A |
| Behavioral performance measures | N/A |

## Acquisition

| | |
|---|---|
| Imaging type(s) | Structural |
| Field strength | 7 Tesla |
| Sequence & imaging parameters | T2 weighted turbo-spin echo sequence (RARE) with 32 axial slices per 0.5 mm, FOV 25.6 x 25.6 mm2, Matrix size 256 x 256, TR/TE = 4200 / 36 ms, 4 avarages, rare factor 8 |
| Area of acquisition | Whole brain |

Diffusion MRI ☐ Used ☒ Not used

## Preprocessing

| | |
|---|---|
| Preprocessing software | Data acquisition and image processing were carried out with Paravision 4.0 software (Bruker). Lesion volume segmentation and quantitation was performed with Analyze 10.0 (AnalyzeDirect Inc.) and OsiriX DICOM Viewer (Pixmeo) . |
| Normalization | N/A |
| Normalization template | N/A |
| Noise and artifact removal | N/A |
| Volume censoring | N/A |

## Statistical modeling & inference

| | |
|---|---|
| Model type and settings | Volumetric measurements of edema - corrected lesion volume |
| Effect(s) tested | N/A |

Specify type of analysis: ☒ Whole brain ☐ ROI-based ☐ Both

| | |
|---|---|
| Statistic type for inference (See Eklund et al. 2016) | N/A |
| Correction | N/A |

## Models & analysis

| n/a | Involved in the study |
| --- | --- |
| ☒ | ☐ Functional and/or effective connectivity |
| ☒ | ☐ Graph analysis |
| ☒ | ☐ Multivariate modeling or predictive analysis |
